# Scalable and Isotropic Expansion of Tissues with Simply Tunable Expansion Ratio

**DOI:** 10.1002/advs.201901673

**Published:** 2019-09-30

**Authors:** Han‐Eol Park, Dongkil Choi, Ji Su Park, Changgon Sim, Sohyun Park, Sunah Kang, Hyunsoo Yim, Myungsun Lee, Jaeyoun Kim, Jinyoung Pac, Kunsoo Rhee, Junho Lee, Yunjong Lee, Yan Lee, Sung‐Yon Kim

**Affiliations:** ^1^ Institute of Molecular Biology and Genetics Seoul National University Seoul 08826 South Korea; ^2^ Department of Biological Sciences Seoul National University Seoul 08826 South Korea; ^3^ Department of Chemistry Seoul National University Seoul 08826 South Korea; ^4^ Division of Pharmacology Department of Molecular Cell Biology Samsung Biomedical Research Institute Sungkyunkwan University School of Medicine Suwon 16419 South Korea

**Keywords:** chemical tissue processing, expansion microscopy, hydrogel‐tissue chemistry, super‐resolution microscopy, tissue expansion

## Abstract

Tissue expansion techniques physically expand swellable gel‐embedded biological specimens to overcome the resolution limit of light microscopy. As the benefits of expansion come at the expense of signal concentration, imaging volume and time, and mechanical integrity of the sample, the optimal expansion ratio may widely differ depending on the experiment. However, existing expansion methods offer only fixed expansion ratios that cannot be easily adjusted to balance the gain and loss associated with expansion. Here, a hydrogel conversion‐based expansion method is presented, that enables easy adjustment of the expansion ratio for individual needs, simply by changing the duration of a heating step. This method, termed ZOOM, isotropically expands samples up to eightfold in a single expansion process. ZOOM preserves biomolecules for post‐processing labelings and supports multi‐round expansion for the imaging of a single sample at multiple zoom factors. ZOOM can be flexibly and scalably applied to nanoscale imaging of diverse samples, ranging from cultured cells to thick tissues, as well as bacteria, exoskeletal *Caenorhabditis elegans*, and human brain samples.

## Graphical abstract

ZOOM, a new tissue expansion technique based on the hydrogel conversion reaction, is developed to enable scalable and isotropic expansion of biological samples with easily tunable expansion ratio (up to eightfold). This method allows for simple and flexible expansion of a wide range of biological samples, from bacteria to human brain tissues, for super‐resolution imaging of samples with ordinary microscopes.

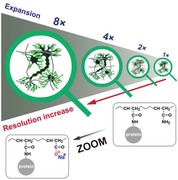

## 1 Introduction

Light microscopy is a principal tool in biology and medicine to obtain detailed molecular and structural information from diverse biological samples. However, the spatial resolution of conventional light microscopy is limited to ≈200 nm by the diffraction of light. Super‐resolution microscopy techniques were developed based on optical and computational approaches to overcome the resolution limit and successfully revealed nanoscale biological structures.^1^, ^2^, ^3^, ^4^, ^5^ Nevertheless, these techniques are not easily scalable to 3D imaging of large tissue samples, owing to slow acquisition speed, photobleaching of fluorophores, optical aberrations at depth, and the requirement for costly and specialized equipment.^6^, ^7^

Emerging tissue expansion techniques—including expansion microscopy (ExM),^8^ magnified analysis of proteome (MAP),^9^ and related protocols^10^, ^11^, ^12^, ^13^, ^14^, ^15^—physically expand biological specimens to enable super‐resolution imaging with conventional diffraction‐limited microscopes.^16^, ^17^ In these methods, a swellable polymer hydrogel is formed throughout a preserved specimen to covalently link key labels or biomolecules, and the resulting hydrogel‐tissue hybrid is fully or partially digested and/or denatured to allow for subsequent expansion in deionized (DI) water. The expanded specimen consists largely of water, rendering the interior optically homogenous and thus transparent. As such, tissue expansion techniques can also facilitate rapid nanoscale imaging across a large volume of transparentized samples when combined with fast volumetric imaging modalities, such as light‐sheet microscopy.^17^, ^18^, ^19^, ^20^, ^21^, ^22^, ^23^, ^24^, ^25^, ^26^ In practice, however, sample expansion enlarges imaging volume and dilutes fluorescence signals cubically with the expansion ratio; these factors synergistically increase imaging time and photobleaching, creating a significant challenge in imaging even with state‐of‐the‐art imaging modalities (Figure S1, Supporting Information).^14^, ^27^, ^28^ Furthermore, excess expansion makes the sample too fragile for handling and stable imaging, and may also increase the sample thickness beyond the working distance limit of the objective lens. Therefore, depending on the experimental goal, one may wish to perform a small, moderate, or large expansion to balance the gain and loss.^29^

An expansion technique in which the expansion ratio can be flexibly adjusted would allow experimenters to conveniently choose the optimal expansion factor based on individual needs. However, currently available methods offer only fixed expansion ratios. In principle, the expansion ratio in any protocol can be adjusted by changing the hydrogel composition and reaction conditions, but extensive optimization and validation may be needed. Alternatively, using a salt‐containing solution at the final expansion step to reduce the expansion ratio has been suggested,^6^ but it can be impractically challenging to precisely maintain the salt concentration of the immersion medium during hours to days of nanoscale imaging of expanded samples, which are highly sensitive to even slight changes in salt concentration. Besides, existing expansion techniques are limited in the expansion ratio, scalability to thick samples, ease of implementation, or a combination of these. The expansion ratios of well‐established protocols are mostly limited to 2‐, 4‐, or 4.5‐fold.^8^, ^9^, ^10^, ^11^, ^12^, ^13^, ^26^, ^29^, ^30^ Recently developed protocols achieve 10‐ or even 20‐fold expansion of samples, but these methods have only been applied to relatively small samples (less than 100 µm thickness),^14^, ^15^ are too complicated for widespread use,^14^ or deform tissue microstructures.^26^

Here, we have developed an expansion technique, which we term ZOOM (an acronym for “Zoom by hydrOgel cOnversion Microscopy”) that enables simple adjustment of the expansion ratio, simply by changing the duration of a heating step. ZOOM also addresses the aforementioned challenges, with improved achievable expansion ratio (eightfold), scalability, and easy protocol steps. We show that ZOOM preserves endogenous biochemical contents as well as the structural organization, supporting post‐processing molecular phenotyping of expanded cells or tissues with conventional antibodies, and multi‐round expansion for the imaging of the same sample at multiple expansion ratios. We also demonstrate the versatility of the technique by applying ZOOM to a wide variety of samples for nanoscale imaging, with minimal adaptations in the protocol for distinct types of samples, demonstrating the versatility of the technique.

## 2 Results

### 2.1 Theoretical Considerations for the Expansion of Hydrogel‐Tissue Hybrids

To develop an expansion method that supports an easy adjustment of the expansion ratio, we considered the primary factors that allow hydrogel‐tissue hybrids to expand. The swelling pressure (π_tot_) of a gel‐tissue hybrid, which competes with the osmotic pressure of the external solution, is mainly determined by the gel osmotic pressure arising from polymeric chains and biomolecules within the hybrid itself (π_mix_), gel‐tissue hybrid network elasticity (π_el_), and mobile ions in the hybrid (π_ion_) (Equation (1))^31^

(1) $$\pi_{\mathrm{tot}}=\pi_{\mathrm{mix}}+\pi_{\mathrm{el}}+\pi_{\mathrm{ion}}$$

Therefore, higher polymer concentrations for more positive π_mix_, lower crosslinking for less negative π_el_, and higher counterion concentrations for more positive π_ion_ would favor larger expansion of gel‐tissue hybrids. As such, in all existing expansion techniques, 1) high‐concentration monomers (mostly acrylamide (AA) or its derivatives) form dense hydrogels across the samples to increase π_mix_, 2) sodium acrylate (SA), an ionic monomer, is incorporated in the gel network to increase π_ion_, and 3) biomolecules are digested, denatured, and dissociated to decrease the degree of crosslinking in the tissue‐hydrogel network, thereby increasing π_el_ (with less negative value).^8^, ^9^, ^10^, ^11^, ^12^, ^14^, ^15^

### 2.2 Hydrogel Conversion‐Based Tissue Expansion Strategy Enables Easy Tuning of the Expansion Ratio

**Figure 1:**
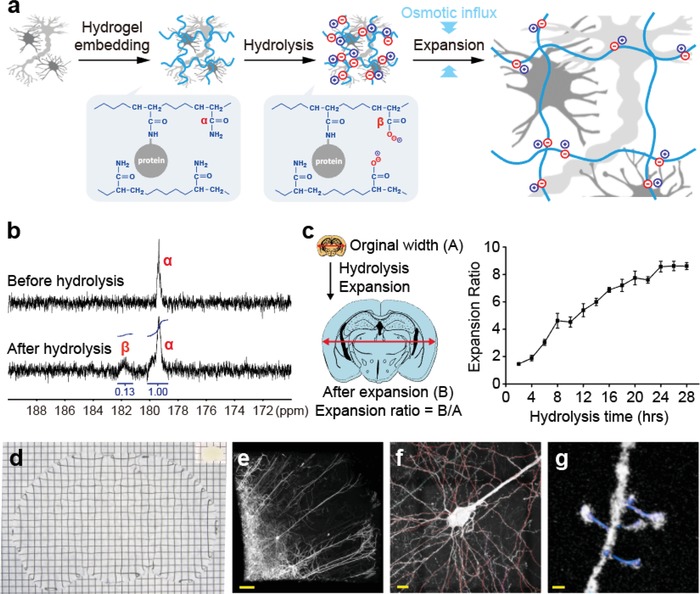
Chemical principles and implementation of ZOOM. a) Schematic illustration of the ZOOM process. A sample is embedded in an electrically neutral acrylamide gel, then undergoes alkaline hydrolysis for the stochastic and uniform introduction of ionic residues throughout the hydrogel network. Note the conversion of nonionic primary amides (α) to ionic carboxylates (β). In this step, biomolecules are partially denatured and dissociated to allow for the subsequent expansion in a low‐osmolality solution. b) ^13^C NMR spectra of polyacrylamide polymers before and after hydrolysis. Note the appearance of a peak corresponding to the carboxylate group (β). After 24 h of hydrolysis reaction at 80 °C, 12% of amides were converted to carboxylates. c) Hydrolysis reaction governs the amount of negative charges introduced in the gel and the degree of sample denaturation, which are the primary factors for determining the expansion ratio. Therefore, the expansion ratio can be easily tuned simply by changing the hydrolysis time. Gel‐embedded 500 µm‐thick mouse brain coronal sections were expanded with increasing hydrolysis times. Expansion ratio scaled with hydrolysis time up to eightfold (*n* = 5). Expansion ratio here was defined as the ratio of sample widths before and after expansion. Data are mean ± s.d. d) A 500 µm‐thick Thy1‐eYFP mouse brain section was eightfold expanded with ZOOM. Photograph of the sample before and after expansion. e) 3D rendering of an expanded cortical tissue volume (immunostained for eYFP following hydrolysis to visualize quenched eYFP molecules) acquired with confocal microscopy (acquired with 10×, 0.5 NA objective lens; acquisition volume, ≈9.0 × 9.0 × 1.8 mm^3^ post‐expansion) f) readily supports tracing of neural processes (red) and g) detection of dendritic spines and necks (blue). Grids, 3.0 mm. Scale bars e) 100 µm, f) 10 µm, g) 500 nm. White scale bars indicate physical dimensions, and yellow scale bars correspond to pre‐expansion dimensions throughout the paper.

To conveniently modulate π_ion_, we sought for a simple chemical approach that enables a tunable introduction of ionic carboxylates, without requiring the preparation of different monomer solutions for incorporating different amounts of ionic residues. Previous investigations have found that alkaline hydrolysis of a polyacrylamide hydrogel stochastically (and therefore uniformly) introduces ionic residues into the gel network by converting nonionic primary amide side chains to carboxylates with counterions, which would increase π_ion_.^32^, ^33^ Coincidentally, this “hydrogel conversion” reaction is facilitated by high pH and heat, which promotes protein denaturation and dissociation to decrease biomolecule‐based crosslinking points, thereby increasing π_el_.^9^, ^11^, ^32^, ^33^ As such, the hydrogel conversion reaction allows for the simultaneous increase in both π_ion_ and π_el_ to synergistically facilitate expansion. A tissue expansion method based on the hydrogel conversion reaction would enable easy tuning of the expansion ratio simply by changing the hydrolysis time, without requiring efforts to optimize hydrogel monomer composition or other reaction conditions to obtain desired expansion factors for individual experiments (**Figure** **1**a). We realized this idea in ZOOM. In this method, a sample is embedded in a high‐concentration (30% w/v) AA gel and then undergoes alkaline hydrolysis with heat for the uniform introduction of ionic residues throughout the hydrogel network. During the alkaline hydrolysis step, biomolecules are partially denatured and dissociated to allow for the subsequent expansion in a low‐osmolality solution. The degree of hydrolysis and sample denaturation, together controlled by the hydrolysis time, would determine the expansion factor.

We first confirmed the changes in molecular identity by alkaline hydrolysis using an inverted‐gate ^13^C NMR spectroscopy. A significant portion of primary amides was converted to carboxylates under high pH and heat after 24 h, as indicated by the downshift of ≈12% of ^13^C signals by 3 ppm (Figure 1b). We then characterized the relationship between the expansion ratio and the hydrolysis time using mouse brain tissues. Remarkably, the expansion ratio, which we refer to as the “ZOOM factor,” exhibited approximately a linear relationship with the hydrolysis time, up to approximately eightfold until 24 h of hydrolysis (Figure 1c). Using this protocol, we were able to expand a 500 µm‐thick coronal section of Thy1‐eYFP mouse brain by eightfold in a single expansion process (4 mm thick after expansion) (Figure 1d–g). Under the conditions leading to eightfold expansion, the brain section became transparent (Figure 1d), while preserving mechanical integrity sufficient for easy handling, post‐processing labeling (to visualize quenched eYFP molecules during the hydrolysis step), mounting, and stable imaging for over 18 h (Figure 1e–g). We note that further hydrolysis can increase the ZOOM factor over 8, but the sample starts to lose structural integrity, becoming too fragile to handle in the following staining and imaging steps. The ZOOM factor indicates the degree of improvement in attainable resolution.^8^, ^9^, ^10^, ^12^, ^14^ In the dataset shown in Figure 1e–g (acquired with 10×, 0.5 NA objective; see Table S1, Supporting Information for sample preparation and imaging conditions for all images), the effective lateral resolution was improved approximately eightfold with the ZOOM factor of 8.0, such that super‐resolution imaging of fine neural processes, dendritic spines, and their necks could be achieved even with a low‐power objective lens (Figure 1f,g, Movie S1, Supporting Information). We also demonstrated that other organs including the liver, kidney, and heart could be expanded with the same protocol without any special optimization for each case (Figure S2, Supporting Information).

### 2.3 Isotropic and Preservative Expansion with Improved Mechanical Properties

**Figure 2:**
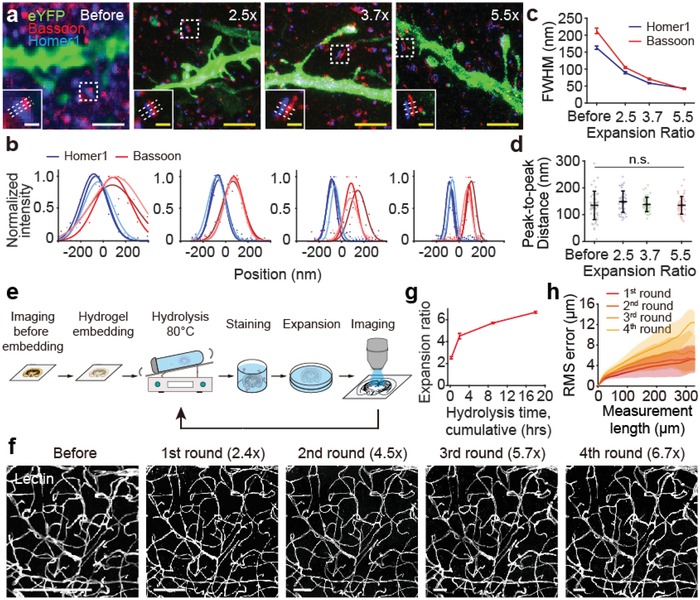
ZOOM enables isotropic expansion while preserving biomolecules for multi‐round expansion. a) Gel‐embedded Thy1‐eYFP mouse brain sections were subject to different hydrolysis times and stained for eYFP (green, a subset of pyramidal neurons), Bassoon (red, a pre‐synaptic marker), and Homer1 (blue, a post‐synaptic marker) to demonstrate improving spatial resolution. b) Intensity plot along the axis of the synapse in insets of (a) showing the gradual separation between pre‐ and post‐synaptic distribution profiles. Intensity values were scaled to 0–1 range by min–max normalization. Lines represent the Gaussian curve fitting. c) Average Gaussian‐fitted FWHM of Bassoon (red) and Homer (blue) profiles decreases sharply upon expansion, indicating enhanced spatial resolution (*n* = 50). d) Peak‐to‐peak distance between Bassoon and Homer profiles remains at ≈150 nm regardless of expansion (one‐way ANOVA) (*n* = 50), indicating preserved structural information. e) Schematic of multi‐round expansion process. A gel‐embedded sample can undergo multiple rounds of hydrolysis (de‐staining at the same time), staining, and expansion while retaining structural integrity. f) A 100 µm‐thick mouse brain tissue was subjected to repeated hydrolysis, staining for blood vessels, expansion, and imaging for four times. Representative images from the same region for each round are shown. g) Increasing hydrolysis time at each round progressively increases the ZOOM factor from 2.4× to 6.7× (calculated during the image registration process, see the Experimental Section for the details). h) RMS error measurement of blood vessel images before ZOOM versus after each round of processing. The estimated distortion (RMS error) was below ≈5% of the measured length (*n* = 4), demonstrating isotropic expansion even after four rounds of hydrolysis at 80 °C. Data are mean ± s.d. Scale bars, a) 2 µm, 400 nm (insets), f) 200 µm.

To investigate the relationship between the ZOOM factor and resolution, we examined closely apposed pre‐ and post‐synaptic proteins (Bassoon and Homer1, respectively) while gradually increasing the ZOOM factor. Bassoon and Homer1 were immunohistochemically labeled following the hydrolysis step, which seems to well preserve epitopes—as demonstrated below with diverse labeling examples and in a related expansion protocol.^9^ We found that the overlapping spots for Bassoon and Homer1 before expansion gradually separated as the ZOOM factor increased to 2.5, 3.7, and 5.5 (**Figure** **2**a,b). The cross‐sectional profile of Bassoon and Homer1 sharpened (Figure 2c) without changes in Bassoon–Homer1 distance (Figure 2d), indicating progressive improvement in resolution while retaining the spatial organization of molecules without detectable distortions. Notably, the width of Homer1, measured as the average Gaussian‐fitted full‐width at half‐maximum (FWHM), could serve as an indicator of the effective imaging resolution (265.9 nm before ZOOM, 94.4 nm at 2.5×, 58.7 nm at 3.7×, and 43.7 nm at 5.5×). The average Bassoon–Homer1 separation was measured to be 146.7 ± 41.3 nm, similar to a previously reported value obtained using the stochastic fluorophore‐switching super‐resolution microscopy (153.4 ± 17.3 nm).^34^ Upon increasing the ZOOM factor, spine necks became precisely detectable without alterations in spine angles (Figure S3, Supporting Information), further supporting improved spatial resolution while preserving structural information.

All existing tissue expansion techniques employ co‐polymerization of AA (or its derivatives) and acrylate (provided in the form of SA) to form the gel‐tissue hybrid with built‐in ionic residues to facilitate expansion.^8^, ^9^, ^10^, ^11^, ^12^, ^13^, ^14^, ^15^, ^25^, ^26^, ^27^, ^30^ In contrast, ZOOM eliminates the use of acrylate for gelation and employs nonionic AA as the only gel monomer. This confers several additional benefits on ZOOM, other than the tunability of the expansion ratio after gelation. The first advantage is the improved chemical uniformity of the hydrogel network within the gel‐tissue hybrid. In principle, compared with ionic acrylates, nonionic AA monomers can rapidly penetrate lipid membranes and thus can be distributed more uniformly among the charged biomolecules.^35^ Furthermore, poor permeability of acrylate ions and sodium counterions into lipid membranes^36^, ^37^ can build up significant osmotic pressure gradient across cellular membranes, even after tissue fixation.^38^ Indeed, we found that high‐concentration SA in the monomer solution (even without high‐concentration sodium chloride that is typically included^8^, ^10^, ^11^, ^14^) caused the tissues to significantly shrink, which can deform cellular morphology and overall tissue structure (Figure S4, Supporting Information). Tissue shrinkage was exacerbated by increasing the proportion of SA while keeping the overall monomer concentration constant. The monomer solution for ZOOM caused negligible distortion, but the solutions for MAP and ExM induced noticeable shrinkage, suggesting that the absence of acrylate may help to alleviate the overall distortion. Besides, since the polymerization rate of acrylate is significantly different from that of AA,^39^ the ionic residue—a key factor for expansion—is unlikely to be evenly distributed on the copolymer network. In contrast, stochastic hydrolysis of the hydrogel constructed by homopolymerization of AA monomers would yield even distribution of ionic residues at the molecular level.

The second advantage of removing acrylate from the hydrogel‐tissue embedding reaction is the improved mechanical properties of the gel. We found that increasing the SA content in the hydrogel monomer solution causes a decrease in the compressive modulus of hydrogel disks, which indicates reduced stiffness (Figure S5a, Supporting Information). Moreover, hydrogels made of high SA‐containing monomer solutions (20–30% w/v) failed to maintain the disk shape after incubation in standard phosphate‐buffered saline (PBS) solution (which slightly expands hydrogel) (Figure S5b, Supporting Information), suggesting that lowering the SA content in monomer solutions would enhance mechanical durability of expanded hydrogels. To test this possibility, we compared the mechanical properties of two expanded gel disks with the matching monomer amounts and expansion ratios, one prepared using a gel‐embedding SA‐containing monomer solution, and the other prepared following the ZOOM protocol (i.e., gel‐embedding AA monomer solution followed by alkaline hydrolysis). Indeed, the gel disks prepared without SA in monomer solutions exhibited better compressive strength as well as toughness (Figure S5c,d, Supporting Information). High toughness and durability of the gel are critical for large expansions, since otherwise expanded samples become too fragile for staining and imaging, and may not even be able to sustain its shape against gravity.^30^, ^40^ Owing to the superior mechanical properties of the gel used in ZOOM, we were able to reduce the crosslinker (bis‐acrylamide) concentration to 0.01% (6–20% of the existing methods^8^, ^9^, ^10^, ^11^, ^12^, ^14^), which contributed to increasing highest attainable expansion ratio, while retaining acceptable mechanical durability in the expanded samples.^8^

Since the gel employed by the ZOOM process provides a stable framework for the hydrogel‐tissue hybrid with favorable mechanical properties, we asked if multiple rounds of ZOOM processing (hereafter “ZOOMing”) is feasible without a significant loss of biological information or tissue integrity, such that the same sample can be imaged multiple times with serial changes in ZOOM factors. To test this, we subjected a gel‐embedded mouse cortical tissue to four rounds of hydrolysis, staining (against blood vessels), and expansion, progressively increasing the cumulative hydrolysis time at each round (Figure 2e). Remarkably, we could successfully acquire images from the same sample with increasing ZOOM factors, from 2.4 to 6.7, without any problem in sample handling (Figure 2f,g). Importantly, the expansion was isotropic, and structural integrity of the tissue was retained even after multiple rounds of ZOOMing; the distortion errors (root‐mean‐square (RMS) distances) estimated by comparing the images before and after ZOOMing were below 5% of the measured length at all rounds (Figure 2h). Taken together, our data demonstrate that ZOOM enables isotropic and preservative expansion of tissues with enhanced mechanical properties.

### 2.4 Nanoscale Imaging of Diverse Subcellular Structures from ZOOMed Cultured Cells

**Figure 3:**
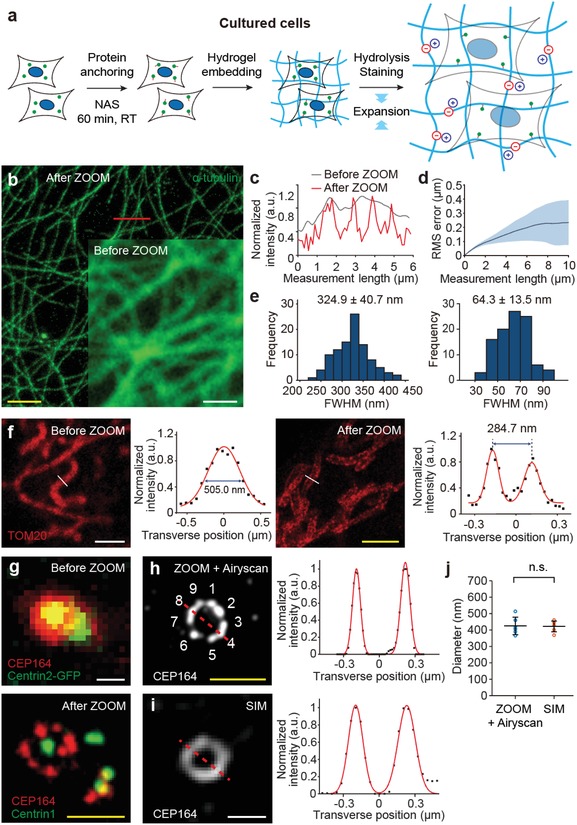
ZOOMing into subcellular structures in cultured cells. a) ZOOM process for cultured cells. b) HeLa cells were stained for α‐tubulin and imaged before and after ZOOM processing for comparisons. Representative confocal images show fine tubulin structures resolved after 4.6× expansion. c) Intensity profiles along the path indicated in (b), before and after expansion. d) RMS error of microtubule images before and after ZOOM processing. The estimated distortion error was less than ≈3% of the measured length (*n* = 4), demonstrating isotropic expansion at the subcellular scale. e) Transverse profiles of 100 microtubules, showing a histogram of FWHM values of Gaussian‐fitted intensity profiles. Average values before (left) and after (right) ZOOM processing indicate improvement in imaging resolution; ≈65 nm width is consistent with the measurement with super‐resolution microscopy and other tissue expansion techniques.^10^, ^41^, ^42^, ^43^, ^44^ f) Mitochondria were stained for TOM20 (a receptor in the mitochondrial outer membrane) and imaged before (left) and after (right) ZOOM processing (3.1× expansion). Transverse profiles of indicated paths (with Gaussian fit) before and after ZOOM show single and double peaks, respectively, demonstrating a clear resolution of the internal space between mitochondrial outer membranes. g) Cells expressing GFP‐centrin2 were stained for CEP164 (centrosomal protein of 164 kDa; labels centriolar distal appendages) before ZOOM processing (top), processed with ZOOM, and stained again for CEP164 and Centrin1 after ZOOM processing (down) (6.3× expansion). h) Individual centriolar appendages can be resolved with ZOOM (imaging with Airyscan feature), i) but not with SIM imaging of nonexpanded samples. j) The average peak‐to‐peak distance of Gaussian‐fitted intensity profiles (indicated dashed lines in (h) and (i)) from six centrioles, corresponding to the longest distance between distal appendages of the centriole structure, was comparable for ZOOM‐processed (425.0 ± 53.4 nm) and nonexpanded SIM‐imaged samples (422.1 ± 32.8 nm) (*p* = 0.8182, Mann–Whitney *U* test). Data are mean ± s.d. Scale bars, b) 5 µm, f) 2 µm, g,h) 500 nm.

Next, we applied ZOOM to a variety of samples, first starting with cultured cells. We noted that pre‐treating fixed cells with *N*‐acryloxysuccinimide (NAS), an amine‐reactive anchor that facilitates crosslinking between peptides and the hydrogel network, significantly improved the staining quality (Figure S6, Supporting Information), hence added this step to the ZOOM protocol for the cell expansion experiments (**Figure** **3**a). We chose NAS as the protein anchor, over methacrylic acid *N*‐hydroxysuccinimidyl ester^10^ or acryloyl‐X^11^ employed in other expansion methods, for its structural similarity with the AA monomer and higher solubility than the other anchors, which would aid in the formation of chemically uniform hydrogel copolymer network. With this protocol, we successfully expanded HeLa cells with several ZOOM factors for super‐resolution imaging of subcellular structures and organelles. ZOOM enabled the observation of detailed 3D microtubule structures and identification of individual fibers from closely located microtubule fibers that were not resolvable before expansion (Figure 3b,c; Movie S2, Supporting Information). Consistent with the isotropic expansion demonstrated with tissues (Figure 2h), ZOOM introduced only minimal distortion to cells after expansion, under 5% of the measurement length at both subcellular (Figure 3d) and multicellular scales, over the ZOOM factors ranging from 1.8 to 6.5 (Figure S7, Supporting Information). We also found that ZOOMing effectively reduced average FWHM of microtubules from 324.9 ± 40.7 nm (mean ± s.d.) to 64.3 ± 13.5 nm (Figure 3e), consistent with the microtubule width measured by super‐resolution microscopy techniques^41^, ^42^, ^43^, ^44^ and other expansion techniques at the comparable expansion ratio.^11^

As a robust component of cells, microtubules are a preferred choice for demonstrating super‐resolution and ExM techniques.^5^, ^8^, ^9^, ^10^, ^11^, ^12^, ^14^, ^15^ However, delicate membrane‐bound proteins or epitopes on multisubunit protein complexes might be susceptible to loss during the expansion process. To test this possibility, we stained ZOOM‐processed cultured cells for a membrane‐bound mitochondrial receptor subunit (TOM20) and proteins of the centriolar complex (Centrin and CEP164) (Figure 3f,g). All these proteins could be clearly labeled and visualized, suggesting that ZOOM preserves proteins of these categories. TOM20 and CEP164 label mitochondrial outer membranes and centriolar distal appendages, respectively; these structures were clearly resolved after ZOOMing, indicated by bimodal distributions of cross‐sectional profiles (Figure 3f,h). Furthermore, individual centriolar appendages could be resolved by ZOOM in combination with Airyscan imaging, allowing for the visualization of characteristic ninefold symmetry of the centriole (Figure 3h). In contrast, this could not be achieved by structured illumination microscopy (SIM)—a well‐established super‐resolution microscopy technique—imaging of nonexpanded samples (Figure 3i). Nevertheless, the “diameter” of the centriolar distal appendages was measured to be comparable in both cases (425.0 ± 53.4 nm vs 422.1 ± 32.8 nm; *p* = 0.8182, Mann–Whitney *U* test), providing additional support for the conclusion that ZOOM does not significantly alter fine subcellular details (Figure 3j). Together, our results demonstrate a successful application of ZOOM to cultured cells with an additional protein‐anchoring step, and further validate the preservative and isotropic expansion of samples using ZOOM.

### 2.5 Nanoscale Imaging of Subcellular and Cellular Features from ZOOMed Neural Tissues

**Figure 4:**
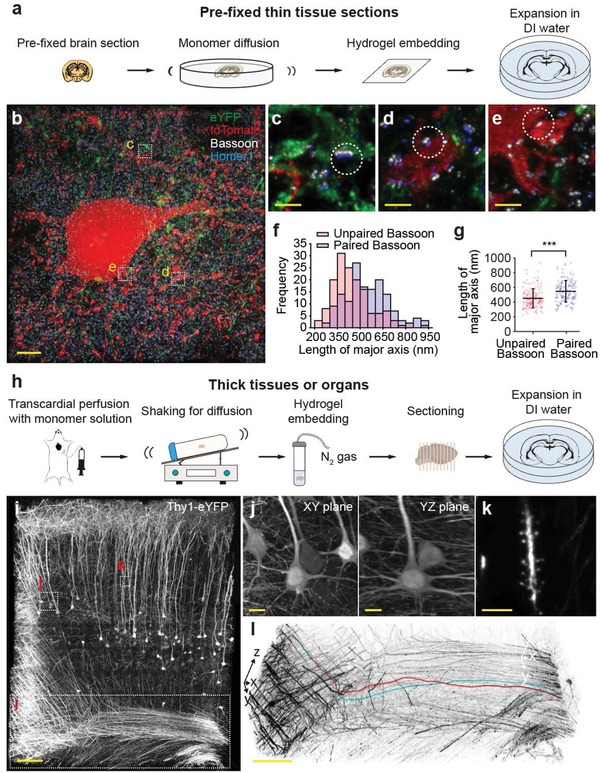
ZOOMing into neural structures in mouse brain tissues. a) ZOOM process for pre‐fixed thin tissue sections. b–g) PV‐tdTomato reporter mice (obtained by breeding PV‐Cre mice with Ai14 reporter line) were injected with adeno‐associated virus carrying eYFP under the CaMKIIα promoter to label parvalbumin‐expressing interneurons with tdTomato (red), and pyramidal neurons with eYFP (green). A formaldehyde‐fixed 100 µm‐thick coronal section from the primary sensory cortex was then subject to ZOOM, with Bassoon (white) and Homer1 (blue) staining (4.0× expansion). b) 3D rendering of cortical tissue area. Bassoon is a pre‐synaptic marker for both excitatory and inhibitory synapses, whereas Homer1 is a post‐synaptic marker for only excitatory synapses. Therefore, an adjacent pair of Bassoon and Homer1 indicates the presence of an excitatory synapse, while unpaired Bassoon may indicate the inhibitory synapse. Consistently, excitatory synapses were found at the c) pyramidal axons and d) dendrites of PV^+^ neurons, while inhibitory synapses were found at the e) synapses between the PV neurons. f) Histogram of major axis lengths of Bassoon paired or unpaired with Homer1 (*n* = 150 for each). g) The major axis length of the unpaired Bassoon is significantly shorter than that of the paired Bassoon (*p* = 5.76 × 10^−9^, Mann–Whitney *U* test). h) ZOOM process for thick samples, for which transcardial perfusion is applicable evenly distributed monomer solutions. i–l) A 500 µm‐thick Thy1‐eYFP mouse brain section was ZOOM‐processed and imaged with light‐sheet microscopy. The whole brain was gel‐embedded after delivering monomers by transcardial perfusion. 5.22 × 10^10^ µm^3^ of ZOOM‐processed mouse cortical tissue (2.42 × 10^8^ µm^3^ before expansion) was imaged with a low‐power objective lens (6.0× expansion). If the unexpanded sample were imaged with a high‐power objective lens to achieve a comparable resolution (e.g., 20×, 1.0 NA objective lens), estimated acquisition time would be 30‐fold longer. i) 3D rendering of the volume image. Despite the poor lens performance, j) the morphology of cell bodies as well as k) dendritic spines is clearly observed at the improved resolution. l) Individual axon fibers could also be readily traced (red and cyan traces). Data are mean ± s.d. Scale bars, b) 5 µm, c–e) 1 µm, i) 100 µm, j) 10 µm, k) 5 µm, l) 100 µm.

We next applied ZOOM to mouse brain tissues and explored the potential of the technique in extracting structural and molecular information at both subcellular and cellular levels.^20^, ^29^, ^34^, ^45^, ^46^ After establishing the ZOOM protocol for pre‐fixed brain tissue sections, a widely available form of brain tissue samples (**Figure** **4**a), we prepared a cortical tissue section where we can visualize excitatory pyramidal neurons, inhibitory PV^+^ interneurons, and synaptic proteins Bassoon and Homer1. To label pyramidal and PV^+^ neurons of the primary somatosensory cortex, we bred a knock‐in mouse line expressing Cre recombinase at the parvalbumin (PV) locus with Cre‐dependent tdTomato reporter (Ai14) mice, and injected adeno‐associated virus expressing eYFP under the control of CaMKIIα promoter into the cortex. We then ZOOMed into the cortical tissue of this mouse by 4.0 times, with staining for Bassoon and Homer1 (Figure 4b). Bassoon is a pre‐synaptic marker for both excitatory and inhibitory synapses, whereas Homer1 is known to be present only at the excitatory post‐synaptic density in the mouse cortex.^46^, ^47^ Therefore, this experimental design allowed us to probe putative glutamatergic and GABAergic synapses (defined here as a pair of partially overlapping Bassoon and Homer1 punctae, or a Bassoon punctae without pairing Homer1, respectively) as well as to visualize a subset of glutamatergic and GABAergic cell bodies and processes. From the ZOOMed cortex, we could identify glutamatergic synapses at the eYFP^+^ glutamatergic axon terminals (Figure 4c) and PV^+^ dendrites (Figure 4d), and GABAergic synapses at the junction of PV axon terminals and PV cell bodies (Figure 4e). Of note, we found that the major axis length of Bassoon paired with Homer1 was significantly longer than that of unpaired Bassoon (*p* = 5.76 × 10^−9^, Mann–Whitney *U* test) (Figure 4f,g), which suggests that the intracellular expression pattern of Bassoon might be different in excitatory and inhibitory terminals.^46^

The combined use of ZOOM with rapid volumetric imaging modalities, represented by light‐sheet microscopy,^17^, ^18^, ^19^, ^20^, ^21^, ^22^, ^23^, ^24^, ^25^, ^26^ may enable rapid high‐resolution imaging across a large volume of tissues. To test this possibility, we transcardially perfused monomer solutions to distribute the monomers throughout the brain, embedded the whole brain, and prepared thick cortical sections (Figure 4h). We then imaged a ZOOM‐processed Thy1‐eYFP mouse brain tissue with a commercially available light‐sheet microscope (Figure 4i). Despite the use of a low‐power objective lens (5×, 0.16 NA), a 6.0× ZOOMing enabled rapid imaging of 2.42 × 10^8^ µm^3^ of the mouse cortex at a resolution sufficient to clearly resolve the morphology of cell bodies (Figure 4j), as well as dendritic spines (Figure 4k) and individual axon fibers (which could also be readily traced; red and cyan traces) (Figure 4l). The acquisition was ≈30 times faster than the estimated time required for standard confocal microscopy imaging of unexpanded samples (with high‐power objectives to achieve comparable resolution), demonstrating that ZOOM may also be used for rapid imaging across a large sample volume.

### 2.6 ZOOMing into *C. Elegans*, Bacteria, and Human Clinical Samples

**Figure 5:**
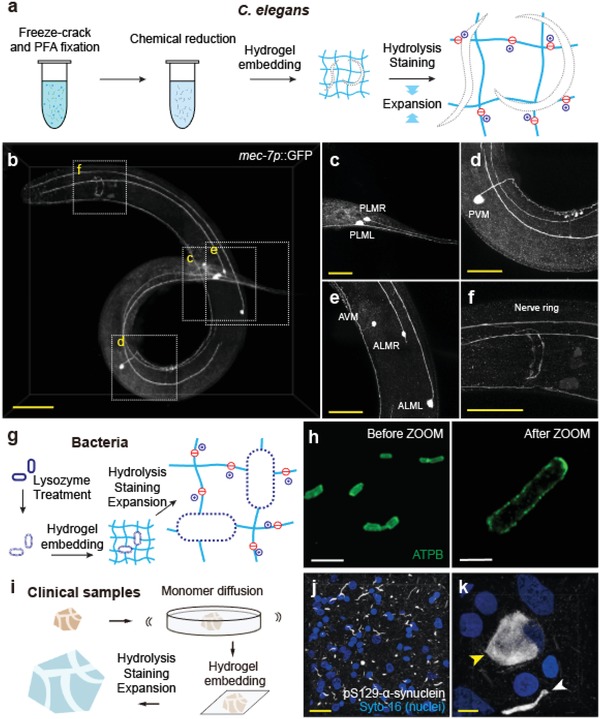
ZOOMing into diverse biological samples. a) Whole transgenic *C. elegans* (*mec‐7p::GFP*) was expanded with modified ZOOM protocol including PFA fixation and β‐mercaptoethanol reduction adapted from tube fixation protocol^48^ and stained for GFP (3.1× expansion). b) Neuronal morphologies were well conserved. c) The PLM neuron cell bodies are located at the lumbar ganglia region, and their main processes run until they reach the midbody region. d) PVM neuron cell body is located at the left side of the posterior body and their processes run along the ventral nerve cord until it reaches to the AVM neuron process. e) The ALM neuron cell bodies are located laterally and their processes run along the lateral sides, whereas the AVM neuron cell body is located at the right site of the anterior body and their processes run along the ventral nerve cord until it reaches to the first bulb of the pharynx. Nerve ring branches of ALML and ALMR processes are shown in (f). g) *E. coli* was expanded using a modified ZOOM protocol, with additional lysozyme treatment for peptidoglycan layer degradation. h) ATP synthase (ATPB) was stained with anti‐ATPB antibody before and after ZOOM processing. i) Formalin‐fixed human brain tissue from a brain bank was expanded with ZOOM. j) A temporal lobe tissue from Parkinson's disease patient was expanded 3.7‐fold and stained for pS129‐α‐synuclein (white) and nucleus (blue). k) Lewy body and Lewy neurite are indicated with yellow and white arrowheads. Scale bars, b) 40 µm, c–f) 20 µm, h) 5 µm, j) 20 µm, k) 5 µm.

Finally, we sought to extend the application of ZOOM to samples that are deemed difficult to be expanded due to their physically and chemically resistant features—exoskeletal nematode *Caenorhabditis elegans*, bacteria *Escherichia coli*, and clinical samples from brain banks. Typically, immunohistochemical staining of *C. elegans* involves chemical reduction and enzymatic degradation of the cuticle, which is mainly composed of collagen‐like proteins.^48^ We partially adopted the histological tube fixation protocol for *C. elegans* and successfully ZOOM‐processed worms (even without enzymatic degradation of cuticle layers by collagenase). Using *mec‐7p::GFP* line, in which touch receptor neurons are labeled with GFP, we found distinct structures of the six *C. elegans* touch receptor neurons (**Figure** **5**a–f). The locations of neurons and their processes were consistent with the previous findings, and the nerve ring branches of the processes of anterior lateral microtubule (ALM) and anterior ventral microtubule (AVM) neurons could be clearly identified.^49^

We also ZOOMed into *E. coli* that has expansion‐resistant peptidoglycan cell walls. We adopted the lysozyme treatment step from standard cell wall‐digesting protocols,^50^ and ZOOM‐processed bacteria (Figure 5g). Rod‐shaped morphology was preserved, as visualized by staining against an inner membrane‐bound protein ATP synthase (ATPB; Figure 5h). Finally, many important clinical samples are preserved in formalin for an extended period of time and thus are heavily cross‐linked. Despite the chemical resistance, the ZOOM protocol for pre‐fixed thin tissue sections (Figures 4a and 5i) successfully expanded formalin‐fixed temporal lobe specimen of Parkinson's disease patient and allowed detailed observation of Lewy body and neurites (Figure 5j,k). Taken together, our results demonstrate the wide applicability of ZOOM to the samples with distinct physicochemical properties.

## 3 Discussion

Tissue expansion techniques enable nanoscale imaging of samples with widely available diffraction‐limited light microscopes.^8^, ^9^, ^10^, ^11^, ^12^, ^14^, ^15^, ^26^ This process can be expedited by rapid imaging modalities, such as light‐sheet microscopy,^17^, ^18^, ^19^, ^20^, ^21^, ^22^, ^23^, ^24^, ^25^, ^26^ or be combined with existing super‐resolution microscopy to further improve the imaging resolution.^23^, ^26^, ^51^, ^52^ As such, tissue expansion methods have an immense potential to impact biological imaging. However, sample expansion is associated with trade‐offs in signal intensity, imaging volume and time, and photobleaching. The low stiffness and durability of the expanded samples create significant problems for further labeling and imaging processes. The thickness of excessively expanded samples may exceed the working distance limit of the objective lens. Therefore, the optimal expansion ratio may differ for individual experiments, bound by the experimental conditions or needs. To fill in this need, we developed ZOOM, in which the expansion ratio can be simply controlled by changing the heating time. Our method increases the utility of tissue expansion techniques to a wider range of biological investigations that may benefit from the expansion of samples with individually suitable expansion ratios.

Easy controllability of the expansion ratio in ZOOM is enabled by employing the hydrogel conversion reaction—in which nonionic primary amide side chains of the AA hydrogel undergoes alkaline hydrolysis to form ionic carboxylate groups—to stochastically introduce ionic residues into the hydrogel network (Figure 1). This is a reaction that has been well characterized and utilized since the 1970s.^32^, ^53^ Simultaneously, biomolecules are dissociated and denatured during the same step. The pH and temperature in the hydrogel‐tissue microenvironment are readily equilibrated throughout a large sample during hydrolysis; thus, compared with the approaches using digestive enzymes to allow for the subsequent expansion,^8^, ^10^, ^11^, ^13^, ^14^, ^15^ ZOOM may be better suited for the scalable processing of samples with various sizes. Higher pH and temperature would facilitate the hydrolysis and denaturation reactions, but we chose mildly basic pH 9 and several temperature steps within the range of 65–95 °C for the hydrogel conversion step, to allow for sufficiently rapid hydrolysis of primary amides, but slow hydrolysis of peptide bonds.^54^ We observed that the resulting gel‐tissue hybrid well retains structural and molecular information that can be extracted with commercially available molecular probes.

In addition to the flexibility in setting the expansion ratio, ZOOM allows for isotropic expansion of large‐scale samples up to eightfold in a single expansion process, without requiring iterative gel embedding and expansion. Recently developed techniques achieve 10‐ or 20‐fold expansion of samples, but these protocols have only been applied to thin sections,^14^, ^15^ involve many chemical steps that are difficult to follow^14^ or causes noticeable deformation in samples.^26^ Other existing methods are simple and scalable, but are limited to 2‐, 4‐, or 4.5‐fold expansions.^8^, ^9^, ^10^, ^11^, ^12^, ^13^, ^26^, ^29^, ^30^ Therefore, ZOOM achieves nearly a twofold improvement in the attainable expansion ratio, compared with the existing expansion methods that are simple and scalable to large samples. This was possible, partly owing to the use of high‐concentration AA gel that provided a mechanically robust framework (also increasing π_mix_) and to enhanced mechanical integrity of the AA‐only gels used in ZOOM, even with a low degree of crosslinking (increasing π_el_) (Figure S5, Supporting Information). Moreover, since the mechanical integrity of gel‐tissue hybrids after the expansion is an important limiting factor in determining the maximum achievable expansion ratio, strategies such as re‐embedding the expanded gel^14^ or developing novel chemical approaches to improve mechanical properties of gel‐tissue hybrid^40^, ^55^ may enable even higher ZOOM factors. The robust mechanical property of the ZOOM‐processed gel‐tissue hybrid also supported multiple rounds of expansion and labeling, such that obtaining images with different ZOOM factors from a single sample was feasible (Figure 2). Multi‐round ZOOMing may potentially be extended to highly multiplexed labeling by performing multicolor labeling at each cycle and overlaying the resulting images, as demonstrated in MAP.^9^

We have shown diverse potential applications of ZOOM, ranging from super‐resolution imaging of subcellular features in cells and tissues^34^, ^45^, ^46^ to rapid high‐resolution volumetric imaging,^19^, ^20^, ^22^, ^23^, ^24^, ^25^, ^26^ demonstrating scalability and versatility of the technique (Figures 3 and 4). Additionally, slight modifications of the basic ZOOM protocol allowed the expansion of adult *C. elegans* with rigid outer cuticle layer, bacteria with cell walls, and heavily fixed human clinical samples, suggesting that ZOOM may also be adapted to expand other diverse kinds of tissues with minimal modifications (Figure 5). The wide applicability of ZOOM, together with its technical advantages and simplicity in implementation, makes itself well poised to accelerate numerous basic and clinical investigations.

## 4 Experimental Section

*Reagents*: Antibodies for immunostaining were purchased as follows: mouse monoclonal anti‐α‐tubulin (T6199, Sigma‐Aldrich), rabbit polyclonal anti‐CEP164 (ab221447, Abcam), mouse monoclonal anti‐Centrin (04‐1624, Merck), mouse monoclonal anti‐ATPB (ab110280, Abcam), rabbit polyclonal anti‐Homer1 (160003, Synaptic Systems), guinea pig polyclonal anti‐Bassoon (141004, Synaptic Systems), goat polyclonal anti‐tdTomato (AB8181‐200, SICGEN), rabbit monoclonal anti‐ribosomal protein S6 (5364S, Cell Signaling Technology), chicken polyclonal anti‐GFP (GFP‐1020, Aves Labs), rabbit polyclonal anti‐GFP (A‐6455, Thermo Fisher Scientific), mouse monoclonal anti‐pS129 α‐synuclein (#825702, Biolegend), Alexa 488‐conjugated rabbit polyclonal anti‐GFP (A‐21311, Thermo Fisher Scientific), Alexa 647‐conjugated rabbit polyclonal anti‐GFP (A‐31852, Thermo Fisher Scientific), Alexa 488‐conjugated rabbit monoclonal anti‐TOMM20 antibody (ab205486, Abcam), Alexa 647‐conjugated rabbit monoclonal anti‐TOMM20 antibody (ab205487, Abcam), Alexa 405‐conjugated donkey anti‐goat IgG (ab175665, Abcam), Alexa 647‐conjugated goat anti‐mouse IgG (ab150115, Abcam), Alexa 647‐conjugated donkey anti‐rabbit IgG (ab150063, Abcam), Alexa 488‐conjugated donkey anti‐rabbit IgG (150061, Abcam), Alexa 594‐conjugated donkey anti‐chicken IgY (703‐585‐155, Jackson ImmunoResearch), Alexa 647‐conjugated donkey anti‐guinea pig IgG (706‐605‐148, Jackson ImmunoResearch), Alexa 647‐conjugated donkey anti‐mouse IgG (A‐31571, Thermo Fisher Scientific).

SYTO‐16 for nuclear staining (S7578) was purchased from Thermo Fisher Scientific. DyLight 488‐labeled tomato lectin (DL‐1174) and DyLight 649‐labeled Tomato Lectin (DL‐1178‐1) were purchased from Vector Laboratories. Normal donkey serum (NDS, 017‐000‐121) was purchased from Jackson ImmunoResearch.

The recombinant AAV vector expressing eYFP (AAV1‐CaMKIIα0.4‐eYFP, 1.2 × 10^13^ copies mL^−1^) was purchased from Penn Vector Core.

High glucose Dulbecco's modified Eagle's medium (DMEM, LM 001‐05), fetal bovine serum (FBS, S 001‐01), 0.1% gelatin solution (LS 023‐01), and Dulbecco's PBS (DPBS, LB 001‐02) were purchased from Welgen.

Sodium chloride (1.06404.1000), Kanamycin (420311‐25GMCN), and glycine (357002) were obtained from Merck Millipore. 4% paraformaldehyde (PFA, P2031) was obtained from Biosesang. Glutaraldehyde (GA, G0068) and AA (A1132) were purchased from Tokyo chemical industry. Piperazine‐*N,N'*‐bis(2‐ethanesulfonic acid) sodium salt (PIPES, PDB0434) was obtained from Bio basic. Ethylenediaminetetraacetic acid disodium salt (EDTA, E5134), magnesium chloride (M8266), bovine serum albumin (BSA, A9647), SA (408220), sodium borohydride (71321), acrylic acid N‐hydroxysuccinimide ester (NAS, A8060), *N,N'*‐methylenebisacrylamide (BA, M7279), *N,N,N',N'*‐tetramethylethylenediamine (TEMED, 411019), ammonium persulfate (APS, 215589), tris(hydroxymethyl)aminomethane (Tris), and dimethyl sulfoxide (DMSO, D5879) were purchased from Sigma‐Aldrich. PFA (32%; 15714) was purchased from Electron Microscopy Sciences. Triton X‐100 (0694) and 2‐mercaptoethanol (βME, 97064) were purchased from VWR Life Science.

*Cell Culture*: HeLa cells were obtained from the Korean Cell Line Bank. To prepare cells for fixation and expansion, 0.1% w/v gelatin‐coated coverslips were placed in 6‐well plates. HeLa cells were then seeded into the wells with the density of 1 00 000 cells per well and incubated for 24 h at 37 °C in DMEM containing 10% v/v FBS and 0.01% w/v kanamycin at 37 °C in the presence of 5% CO_2_. eGFP‐centrin‐expressing HeLa cells were prepared by transfection of HeLa cells (2.4 × 10^5^ cells on a 60 mm dish) with 2.5 µg of the plasmid (eGFP‐Centrin2, Accession number NM_004344.2, pLVX‐IRES‐Puro) using Fugene HD (Promega, E2311). One day after transfection, the cells were transferred to a 100 mm dish and treated with 1 mg mL^−1^ puromycin (Millipor sigma, 540222) for 2–3 weeks and then monoclonal cell lines were established with the dilution cloning method.

*E. coli 25922* cells were obtained from ATCC. Stocks were prepared by adding 75% glycerol 20 µL to *E. coli* 80 µL and stored at −80 °C. For experiments, stocked cells were defrosted and 5 µL of the stock was added to 8 mL of cation‐adjusted Mueller Hinton II Broth (BD), then incubated for 16 h under shaking at 37 °C until OD_600_ is reached to 0.5.

*Animals*: All experimental protocols were approved by the Seoul National University Institutional Animal Care and Use Committee. All mice were housed in a temperature‐ and a humidity‐controlled room with a reverse 12 h light/dark cycle, with ad libitum access to chow food and water. Both male and female mice at least 6 weeks of age were used. C57BL/6J was obtained from DBL. B6.129P2‐Pvalb^tm1(cre)Arbr^/J (PV‐Cre; JAX stock No. 017320) and *Gt(ROSA)26Sor^tm14(CAG‐tdTomato)Hze/J^* (Ai14 mice; JAX stock No. 007914) mice were obtained from the Jackson Laboratory. B6.Cg‐Tg(Thy1‐YFP)HJrs/J (Thy1‐YFP‐H mice; JAX stock no. 003782) were generously provided by Pilhan Kim (Korea Advanced Institute of Science and Technology). To obtain PV‐tdTomato reporter mice, PV‐Cre mice were crossed with Ai14 mice to reveal expression patterns.

*Stereotaxic Surgery*: PV‐tdTomato reporter mice were anesthetized with 1.5–3.0% isoflurane and placed in a stereotaxic apparatus (David Kopf Instruments) while resting on a heating pad. Following a scalp incision, a small craniotomy was made using a hand drill at the regions of interest. 500 nL of AAV was injected to the primary somatosensory cortex using a pressure injection system (Nanoliter 2000) with a pulled glass capillary at 40–100 nL min^−1^. The coordinate was ±0.5 mm antero‐posterior (AP), ±0.5 mm medio‐lateral (ML), −0.5 mm dorso‐ventral (DV) (four injection sites per mouse). The incision was closed using suture and tissue adhesive (Vetbond) and mice were provided with antibiotics and analgesics. Mice were placed in a clean cage on a heating pad to recover from anesthesia, and were kept in their home cage for 3–4 weeks for viral expression and recovery from surgery before transcardial perfusion.

*Mouse Perfusion*: Mice were anesthetized using isoflurane and perfused transcardially with 20 mL of 1 × PBS and 20 mL of fixative solution (4% PFA in PBS or 30% AA and 4% PFA in PBS) at 4 °C. Brains were then harvested and incubated overnight in the same fixative solution at 4 °C with gentle shaking. Brains were sectioned to 50 or 100 µm‐thick coronal slices using a vibrating microtome and stored in 1 × PBS at 4 °C until use.

*C. elegans Culture*: For visualizing touch receptor neurons, the strain CF702 muIs32 [*mec‐7p::GFP + lin‐15(+)*] was obtained from the Caenorhabditis Genetics Center (Strain CF702). Worms were grown at 20 °C on nematode growth media plates with *E. coli* (OP50) bacteria as a food source and handled with the standard methods.^56^ The bleaching technique was used for synchronizing the developmental stages of *C. elegans* at a young adult stage.

*Postmortem Human Brain Tissue*: Fixed postmortem human brain sections (temporal lobe) were kindly provided by Brain and Body Donation Program. Fixed brain sections were subjected to ZOOM and immunostaining as described. The information of the brain section used in this study is as follows: donor ID #, 869; sex, female; age, 79; race, white; PMI, 3; postmortem sections from PD patient with dementia, Lewy‐body‐positive.

*NMR Analysis*: A monomer solution was prepared with 10% w/v AA, 0.1% w/v APS, and 0.1% w/v TEMED in PBS. Polymerization of the solution was conducted under N_2_ atmosphere with mild stirring for 6 h at room temperature (RT). The polymer solution was lyophilized then the obtained solid products were dissolved in basic detergent solution (200 × 10^−3^ m sodium dodecyl sulfate (SDS), 50 × 10^−3^ m boric acid in DI water, pH titrated to 9.0) for alkaline hydrolysis. After 24 h of incubation at 80 °C, the product solution was then purified with dialysis (2 mL product solution in 3.5 kD dialysis bag) against 2 L of DI water. The dialyzing DI water was changed to fresh ones every 12 h for 7 days. The purified product solution was lyophilized and dissolved in D_2_O, and then ^13^C NMR spectra were measured with Agilent 400‐MR DD2 Magnetic Resonance System (400 MHz) at probe temperature in D_2_O. The scan number was 2000 and relaxation delay was 25 s with inverse gated decoupling.

*Hydrogel Embedding*: For embedding cultured cells, cells were fixed for 10 min at RT in a solution containing 3.2% w/v PFA and 0.1% w/v GA either in PEM buffer (0.1 m PIPES, 1 × 10^−3^ m EDTA, 1 × 10^−3^ m MgCl_2_) for microtubule experiments, or in PBS buffer for all other experiments. In case of microtubule fixation, cells were pre‐treated with an extraction buffer (0.5% w/v Triton X‐100 in PEM buffer) for 30 s at RT, to wash out tubulin monomers. After the fixation, cells were incubated in 0.1% w/v sodium borohydride solution for 7 min and in PBS buffer containing 100 × 10^−3^ m glycine for 10 min, to inactivate glutaraldehyde. The cells were then washed three times for 5 min each with PBS and moved to the anchoring solution (25 × 10^−3^ m NAS in 60% v/v DPBS and 40% v/v DMSO). After 60 min of anchoring at RT, the cells were washed three times for 5 min each with PBS. Then the cells were incubated in a monomer solution (30% w/v AA and 0.01% w/v BA in PBS buffer) for 60 min at RT. Finally, the cells were loaded on a coverslip and incubated in 100 µL of monomer solution with initiators (0.5% w/v TEMED and 0.5% w/v APS). The sample was then sandwiched by another coverslip, with polytetrafluoroethylene film (ASF‐110FR, Chukoh) placed in‐between as a spacer. Gelation proceeded at RT for 10 min, followed by brief washing of the gel with PBS buffer.

For embedding pre‐fixed brain sections, 50 or 100 µm‐thick fixed tissue samples (including fixed mouse and human brain samples) were incubated in a monomer solution (30% w/v AA, 0.01% w/v BA, 0.65 m sodium chloride, and 4% w/v PFA in PBS; ZOOM solution) with 0.1% w/v TEMED for 3 h at RT. The sample was moved to a wide No. 1.5 coverglass, and freshly prepared ZOOM solution with 0.1% w/v TEMED and 0.1% w/v APS was added on top of the section. The sample was then sandwiched with another coverglass, No. 1 coverglass as a spacer, to obtain flat hydrogel product. Gelation proceeded for 40 min at 25 °C, and the resulting gel was briefly washed with PBS.

For embedding mouse tissues and organs, mice were first perfused transcardially with PBS and the ZOOM solution with 0.1% w/v V‐50 azo initiator. Brains were then harvested and incubated in a freshly prepared ZOOM monomer solution at 4 °C for 3 days for post‐fixation and monomer equilibration with gentle shaking. After incubation, tissues were moved to 5 mL of a freshly prepared ZOOM solution with 0.1% w/v V‐50. Gel embedding was performed under nitrogen gas at 45 °C for 40 min with gentle shaking, using Easy‐Gel (LifeCanvas Technologies). The gel‐embedded samples were carefully taken out from a tube, and then the excess gel was manually removed. 300–1000 µm coronal sections were obtained from the embedded gel‐tissue hybrid as needed using a vibrating microtome.

For processing *C. elegans*, worms were first fixed and collagen walls were chemically reduced with the tube fixation protocol without collagenase treatment.^48^ Briefly, adult worms were fixed with 4% w/v PFA at 37 °C for 2 h. Worms were then washed in PBS with 0.1% w/v Triton X‐100 (PBST) three times and incubated in a solution containing 5% β‐mercaptoethanol, 1% Triton X‐100, and 0.1 m Tris (pH 7.0) at 37 °C for 12 h with gentle shaking. Following rinsing eight times with PBST, worms were incubated in ZOOM solution with 0.1% w/v TEMED for 12 h at 4 °C with gentle shaking. Worms were then moved to a freshly prepared ZOOM solution with 0.1% w/v TEMED and 0.1% w/v APS. The solution containing worms was dropped on No. 1 coverglass, which was sandwiched with another coverglass with spacers at the edges. Gelation proceeded at 25 °C for 30 min, and the resulting gel was washed with PBS.

For embedding *E. coli, E. coli* 25922 cells were fixed in a Karnovsky's fixative solution for 2 h at 4 °C. After brief washing with PBS, fixed cells were incubated in a lysozyme buffer (940 units mL^−1^ lysozyme, 20 × 10^−3^ m Tris‐HCl, 2 × 10^−3^ m EDTA, 1% Triton X‐100) for 15 min at RT for cell wall digestion. Cells were then washed in PBS and gel‐embedded in the same manner as cultured cells.

*Hydrogel Conversion*: Hydrogel‐embedded samples were first incubated in a heated, basic detergent solution (200 × 10^−3^ m SDS, 50 × 10^−3^ m boric acid in DI water, pH titrated to 9.0) at 95 °C for 15–30 min (depends on sample thickness) for partial denaturation of biomolecules. This was followed by incubation at 80 °C for 0–48 h depending on the desired ZOOM factor. After the conversion, samples were washed four times for 1–2 h each in PBS with gentle shaking.

*Immunostaining*: See Table S1 (Supporting Information) for the list of antibodies used for each experiment. For the immunostaining of cultured cell and *E. coli*, cells were incubated in blocking buffer (3% w/v BSA in PBST) for 30 min at RT. The cells were then incubated in a primary antibody solution (typically diluted at 1:200 to 1:300 in blocking buffer) for 4 h, and washed three times for 5 min each with a blocking buffer. Cells were then incubated in a secondary antibody solution (typically diluted at 1:300 to 1:500 in blocking buffer) for 2 h and finally washed three times for 5 min each in PBST.

For fixed brain sections, free‐floating 50 or 100 µm sections were incubated for 1 h in a blocking buffer (4% NDS in PBST). Sections were incubated with a primary antibody solution at 4 °C for 8–16 h, followed by washing three times for 1 h at RT with PBST. Sections were then incubated with a secondary antibody solution at RT for 3–6 h, followed by washing with PBST for 1–2 h at RT for three times.

For ZOOM‐processed samples, samples were first incubated in blocking buffer at 4 °C for 12–24 h. The samples were incubated with a primary antibody solution (typically 1:100 in PBST) at 4 °C for 1–3 days, followed by washing for 1–4 h in PBST at RT for three times. The tissue was then incubated with a secondary antibody solution (typically 1:100 in PBST) at RT for 1–3 days, followed by washing for 1–4 h at RT with PBST for three times.

*Expansion, Mounting, and Imaging*: The stained HeLa cells on a coverslip were mounted on a microscope slide with PBS as a mounting medium. For brain sections, free‐floating sections were mounted on microscope slides with PVA‐DABCO. Confocal images were obtained on a Zeiss LSM 880 laser scanning microscope. SIM images were collected using the DeltaVision OMX SR imaging system (GE Healthcare, Buckinghamshire, UK).

ZOOM‐processed samples were moved to a petri dish and the dish was filled with DI water. Water was exchanged every 1 h until the sample expansion reached equilibrium. After the expansion is complete, water was carefully removed from the petri dish. Several No. 1 coverglass or small magnets (D101‐N52, K&J Magnetics) were used to build spacers, and a coverslip was laid on top of the sample. To firmly hold the sample in place, 1% agarose gel was formed in the dish fill the spaces between the petri dish and the coverslip. The entire dish was filled with DI water, and samples were imaged using a Zeiss LSM 880 laser scanning microscope.

*Expansion Factor Measurement*: To evaluate the expansion factor for the experiments shown in Figures 1c–g and 4i–l, the length of the long axis of the expanded sections was divided by that of unexpanded sections. For these experiments, whole mouse brains were processed with ZOOM as described above and sectioned to 500 µm‐thick coronal sections with a vibrating microtome in the air to prevent expansion in PBS. The original length of the long axis of the section was measured, and then sections were subject to hydrogel conversion for varying amounts of time. Sections were washed with DI water for three times, and the lengths of the long axis of expanded sections were measured.

For the experiments shown in Figures 2a–d and 4b–g, the expansion factor was calculated as the cube root of the volume ratio between the samples before and after ZOOMing. For the *C. elegans* experiments, the ratio between the average diameters of developmentally synchronized worms, before and after ZOOMing, was used to estimate the expansion factor.

For the experiments shown in Figures 2f–h and 3 and Figure S7 (Supporting Information), the scaling factor of rigid transformation between the images of samples before and after ZOOMing was taken as the expansion factor (see below “Measurement error quantification” section).

*Measurement Error Quantification*: RMS error was estimated in a similar manner with the previous studies.^10^ Briefly, pre‐ and post‐expansion images were converted to 16‐bit grayscale format with Fiji and post‐expansion images were registered to the pre‐expansion images by similarity (rigid) transformation using Elastix software. During the similarity transformation, post‐expansion images were isotropically translocated, rotated, expanded, or contracted to match the corresponding positions of the pre‐expansion images, and the scaling factor was computed through the transformation processing. The resulting, transformed images were again registered to pre‐expansion images by B‐spline (nonrigid) transformation using Elastix to determine the distortion. The output data were then processed using Mathematica scripts provided by a previous report^10^ to generate the measurement RMS error plots. In these data, RMS error was calculated from all combinations of random sampled 7000 input points.

*Line Profile Intensity and FWHM Analysis*: To analyze the line intensity profiles, straight lines were drawn perpendicular to the synaptic junction or near the fibers of interest and intensity profiles were obtained. Signals from individual channels in profiles were normalized by Min‐Max scaling. 1D Gaussian distributions were fit to normalized signal intensity and FWHM was measured using Matlab.

*Neurofilament Tracing*: Individual neurons were semi‐automatically traced using filament tool of Imaris software (Bitplane). An image of the mouse cortex was loaded into a 3D view, and the “autopath” calculation was performed by selecting individual cell body as a starting point, and neuronal fibers were designated by selecting endpoints of connected volume. Dendritic spine tracing was performed in a similar manner using autopath calculation.

*Tissue Shrinkage Test*: 4% w/v PFA fixed 1 mm thick brain slices were incubated following monomer solutions without initiators: 30% w/v AA, 10% w/v SA, 0.05% BA, and 1 × PBS (original MAP); 2 m NaCl, 2.5% w/w AA, 8.625% w/w SA, 0.15% w/w BA, and 1 × PBS (ExM); 0.6 m NaCl, 30% w/v AA, 0.01% w/v BA, and 1 × PBS (ZOOM; AA30); 20% w/v AA, 10% w/v SA, 0.01% w/v BA, and 1 × PBS (AA20 SA10); 10% w/v AA, 20% w/v SA, 0.01% w/v BA, and 1 × PBS (AA10 SA20); 30% w/v SA, 0.01% w/v BA, and 1 × PBS (SA30). Relative areas were calculated based on area of coronal section before and after incubation, measured with Fiji.

*Compressive Strength Measurement*: To evaluate the mechanical properties of cylindrical hydrogel disks made of different compositions, Galdabini Quasar 5 universal testing machine was used to measure the strain‐compressive strength relationship. Gel disks were placed between the fixed lower plate and the moving upper plate, which was connected to a load cell (250 N) with a crosshead. The moving plate pressed the hydrogel at the speed of 5 mm min^−1^, while plate movement and force on the load cell was recorded. The strain was defined as the relative change in the disk thickness, and the strength was calculated as the pressure, based on measured force and the initial cross‐sectional area of gel disks.

*Statistics and Reproducibility*: All experiments were performed at least three times independently unless indicated otherwise. All data are expressed as the mean ± s.d. *n* values are stated in figure legends. One‐way analysis of variance (ANOVA) was used as indicated in the figure legends.

## Conflict of Interest

The authors declare no conflict of interest.

## Supporting information

Supplementary

Supplementary

Supplementary
