# Supplementary material for: Scalable and Isotropic Expansion of Tissues with Simply Tunable Expansion Ratio
Source: Adv Sci (Weinh). 2019 Sep 30;6(22):1901673. doi: 10.1002/advs.201901673 (PMC6864509; doi:10.1002/advs.201901673)
Supplement: Supplementary file 1 — Supplementary [file ADVS-6-1901673-s002.pdf]

Copyright WILEY-VCH Verlag GmbH & Co. KGaA, 69469 Weinheim, Germany, 2019.

ADVANCED SCIENCE
Open Access

Supporting Information

for *Adv. Sci.,* DOI: 10.1002/advs.201901673

Scalable and Isotropic Expansion of Tissues with Simply Tunable Expansion Ratio

Han-Eol Park, Dongkil Choi, Ji Su Park, Changgon Sim, Sohyun Park, Sunah Kang, Hyunsoo Lim, Myungsun Lee, Jaeyoun Kim, Jinyoung Pac, Kunsoo Rhee, Junho Lee, Yunjong Lee, Yan Lee, and Sung-Yon Kim**

Copyright WILEY-VCH Verlag GmbH & Co. KGaA, 69469 Weinheim, Germany, 2019.

Supporting Information

Scalable and Isotropic Expansion of Tissues with Simply Tunable Expansion Ratio

Han-Eol Park, Dongkil Choi, Ji Su Park, Changgon Sim, Sohyun Park, Sunah Kang, Hyunsoo Lim, Myungsun Lee, Jaeyoun Kim, Jinyoung Pac, Kunsoo Rhee, Junho Lee, Yunjong Lee, Yan Lee and Sung-Yon Kim**

H. -E. Park,[+] J. S. Park, C. Sim, H. Lim, M. Lee, Prof. J. Lee, Prof. S. -Y. Kim
Institute of Molecular Biology and Genetics, Seoul National University, Seoul 08826, South Korea
E-mail: gacn@snu.ac.kr; sungyonkim@snu.ac.kr

H. -E. Park, H. Lim, J. Kim, Prof. K. Rhee, Prof. J. Lee
Department of Biological Sciences, Seoul National University, Seoul 08826, South Korea

D. Choi,[+] C. Sim, S. Park, S. Kang, M. Lee, J. Pac, Prof. Yan Lee, Prof. S. -Y. Kim
Department of Chemistry, Seoul National University, Seoul 08826, South Korea

Prof. Yunjong Lee
Division of Pharmacology, Department of Molecular Cell Biology, Samsung Biomedical Research Institute, Sungkyunkwan University School of Medicine, Suwon 16419, South Korea

[+]These authors contributed equally to this work.

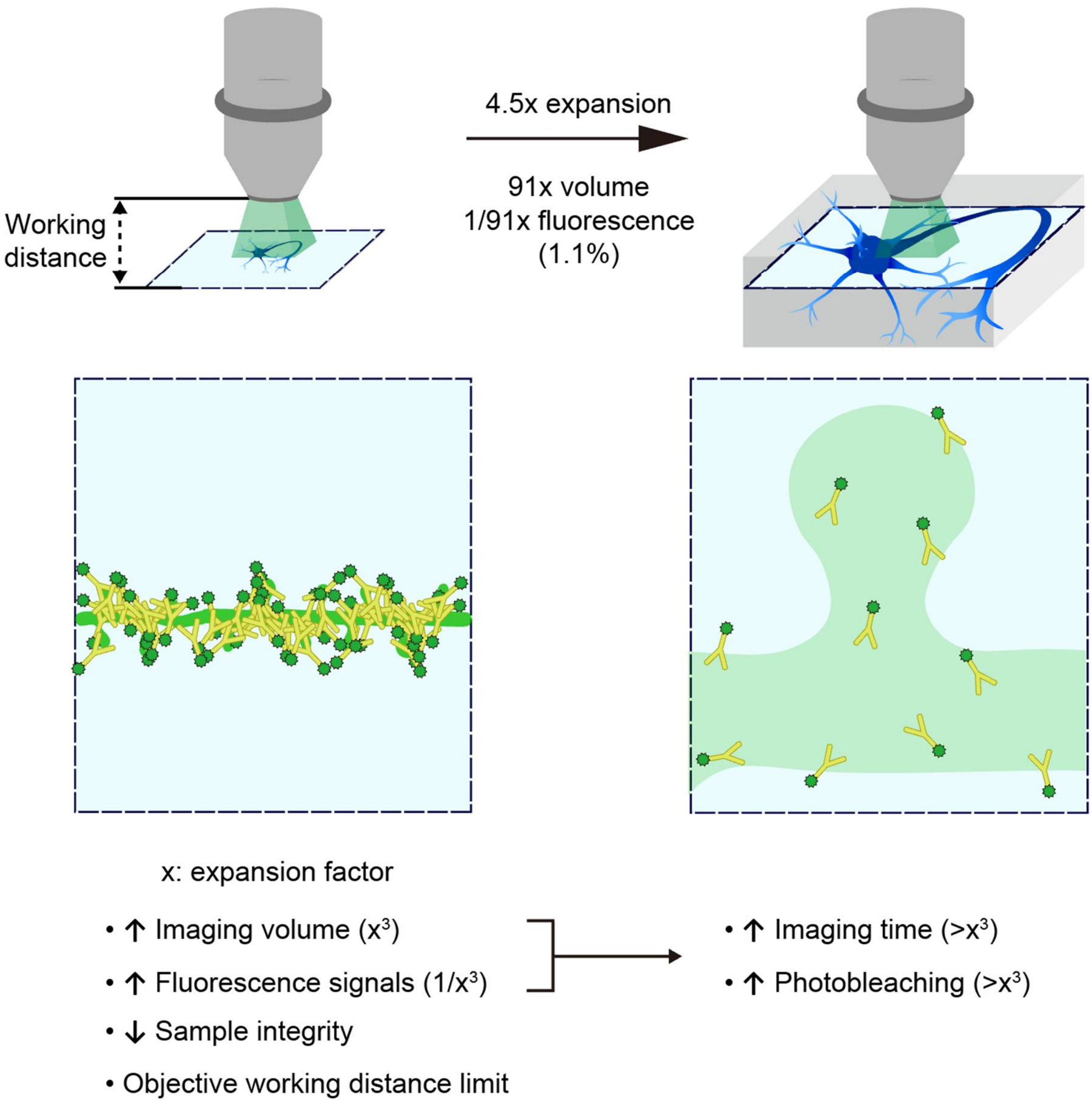

Figure S1. Disadvantages of sample expansion.

An expansion of a biological sample at the linear expansion ratio 'x' leads to an x^3-fold increase in imaging volume and x^3-fold decrease in the fluorophore concentration. Diluted fluorophore concentration necessitates a compensatory increase in the excitation light power and/or pixel dwell time for imaging at a comparable signal-to-noise ratio, both of which aggravates photobleaching. These factors also increase the imaging time equal to or larger than x^3-fold. The expansion also renders mechanical rigidity of the samples weak, and the thickness of the expanded samples may exceed the working distance limit of the objective lens.

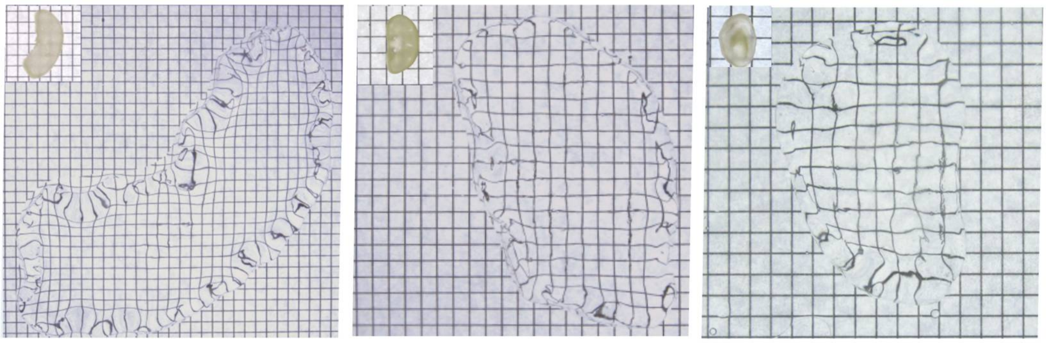

Figure S2. Application of ZOOM to mouse organs.

500-μm thick sections of mouse liver (left), kidney (middle), and heart (right) were ZOOM-processed, with the hydrolysis time of 18 hrs. Each organ shows a similar but different expansion factor due to different tissue compositions. ZOOM factors: 5.2 for liver, 5.9 for kidney, 5.0 for heart. Grids, 3.0 mm.

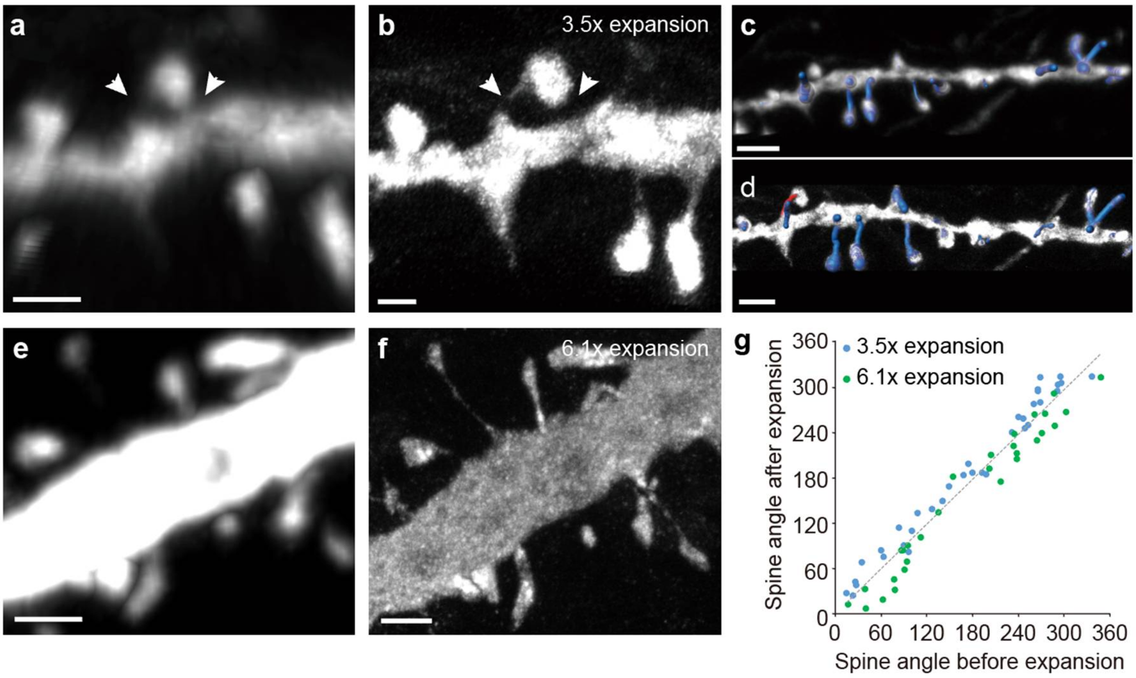

Figure S3. Increasing the ZOOM factor enhances spatial resolution while preserving structural information of dendrites and spines.

a,b) Dendritic spines before (a) and after (b) 3.5-fold expansion. Note the existence of the neck on the left arrowhead can be clearly determined after processing the tissue with ZOOM. c,d) Tracing spines before and after expansion. e,f) Dendritic spines from another sample before (e) and after (f) 6.1-fold expansion. g) Spine angles were overall maintained after ZOOM processing with two different ZOOM factors. Linear regression, y = 0.986x + 0.917, R^2 = 0.9489, p = 9.18e-42. n = 64 (29 from 6.1x sample and 35 from 3.5x sample). Scale Bars, 1 µm (a), 2 µm (b,c,e), 5 µm (d,f).

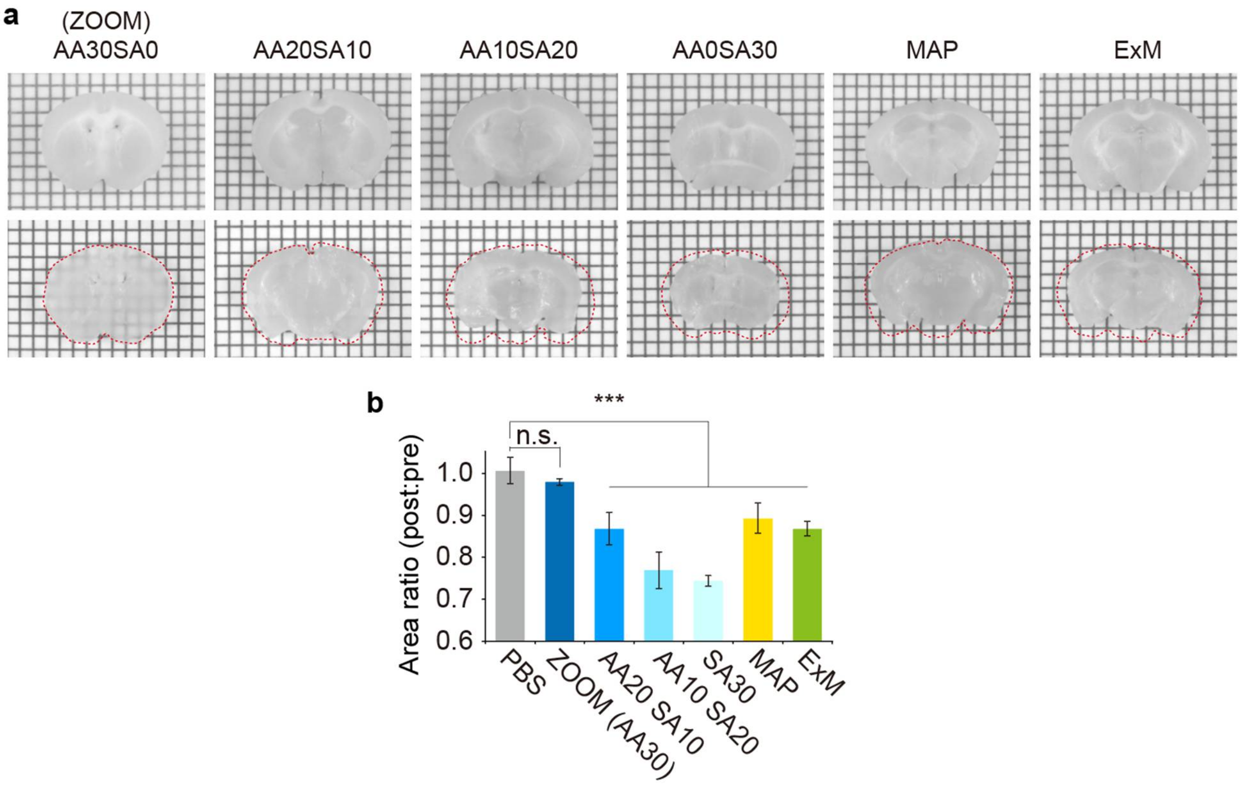

Figure S4. High-concentration sodium acrylate in monomer solutions distorts tissue samples. Fixed tissue sections were incubated in an array of monomer solutions for 1 hr. Representative images (a) and quantification of tissue shrinkage (b), showing that increasing the proportion of sodium acrylate, a key monomer component in all existing expansion methods, causes tissues to shrink (*n* = 4 sections from 2 mice). Y-axis indicates the ratio of the pre-expansion area (inside red dotted lines) to the post-expansion area. Note that the monomer solutions for MAP or ExM also significantly distort the tissue (One-way ANOVA with the *Bonferroni post-hoc* test, P-value for each condition: 1 (ZOOM), 3.60×10^{-5} (AA20 SA10), 4.73×10^{-9} (AA10 2A10), 7.40×10^{-10} (SA30), 5.15×10^{-4} (MAP), and 3.52×10^{-5} (ExM), all versus PBS). AA, acrylamide; SA, sodium acrylate. Numbers indicate weight/volume% concentration of each chemical in monomer solutions. Grids, 1.0 mm. Data are mean ± s.d.

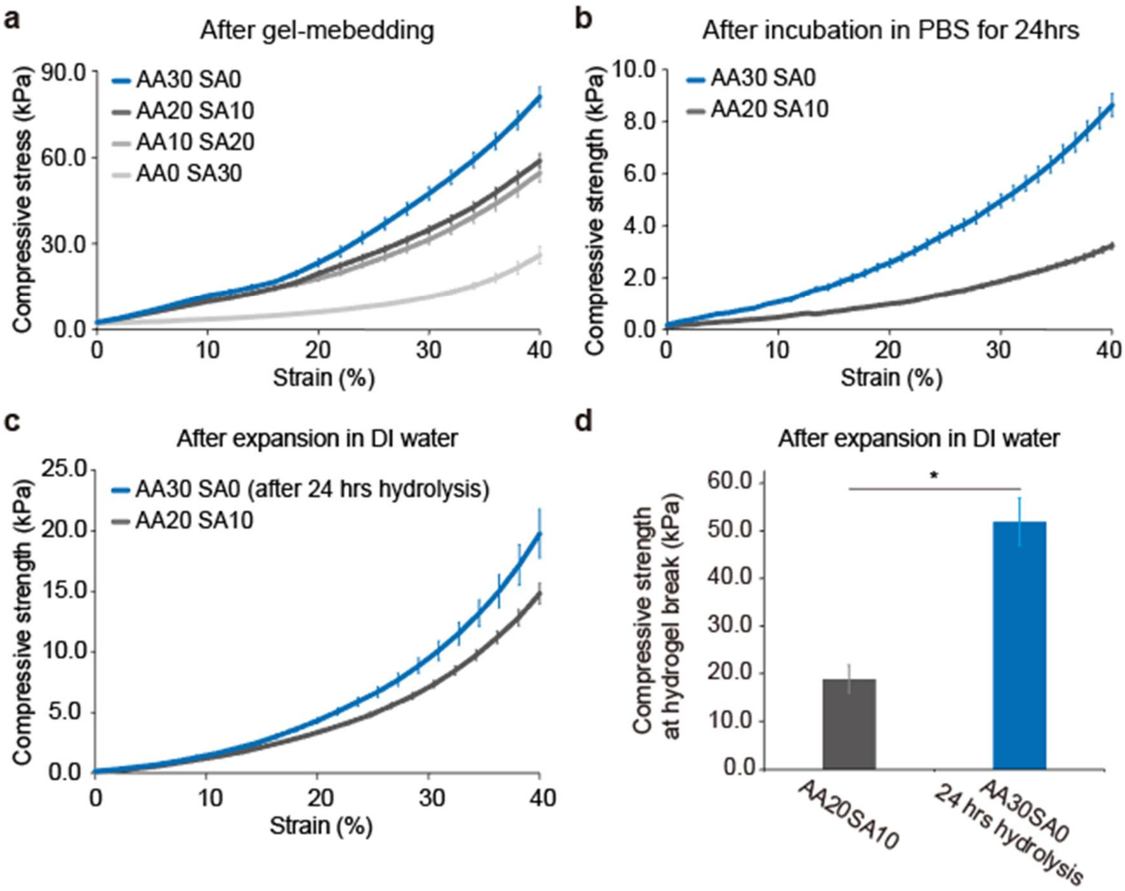

Figure S5. Increasing sodium acrylate content in the acrylamide hydrogel degrades mechanical properties.

To measure the stress-strain relationship of cylindrical hydrogel discs made of various acrylamide (AA) and sodium acrylate (SA) contents, gel discs were made in a 24-well plate with indicated percentage of AA and SA, 0.01% *N,N′*-methylenebisacrylamide, 0.1% APS, and 0.1% TEMED (all in % w/v). a) Compressive stress was applied to cylindrical hydrogel after gel embedding, and the resulting strain was measured with a universal testing machine. Stiffness of the gel discs gradually deteriorated as the SA content increased (n = 6). b) Compressive stress versus strain measured after incubation in 1× PBS for 24 hrs, again demonstrating decreased gel rigidity upon increasing SA contents (n = 6). Compressive stress-strain properties could not be measured from gel discs made of equal or more than 20% of SA, because these discs failed to maintain the shape after incubation in PBS. Gel discs mildly but not fully expand in PBS, due to the high salt contained in the solution. c) Two gel discs made of 30% monomers were investigated. One disc was prepared by co-polymerizing 20% AA and 10% SA, and the other disc was prepared by 30% AA only, followed by alkaline hydrolysis in 24 hrs. Both discs were incubated in DI water for expansion, and compressive properties were measured. Although the hydrolyzed AA30 SA0 discs expand to the similar or

larger extent than AA20 SA10 discs (data are not shown), AA30 SA0 discs exhibited higher stiffness than AA20 SA10 discs. d) The measured compressive strength applied to the samples in c when samples were broken (n = 4). Therefore, AA30 SA0 hydrogel discs after alkaline hydrolysis had significantly better resistance to compressive force than AA20 SA10 discs under the conditions leading to comparable expansion ratios, indicating higher toughness and durability (p = 0.0286, Mann-Whitney U test). Data are mean ± s.e.m.

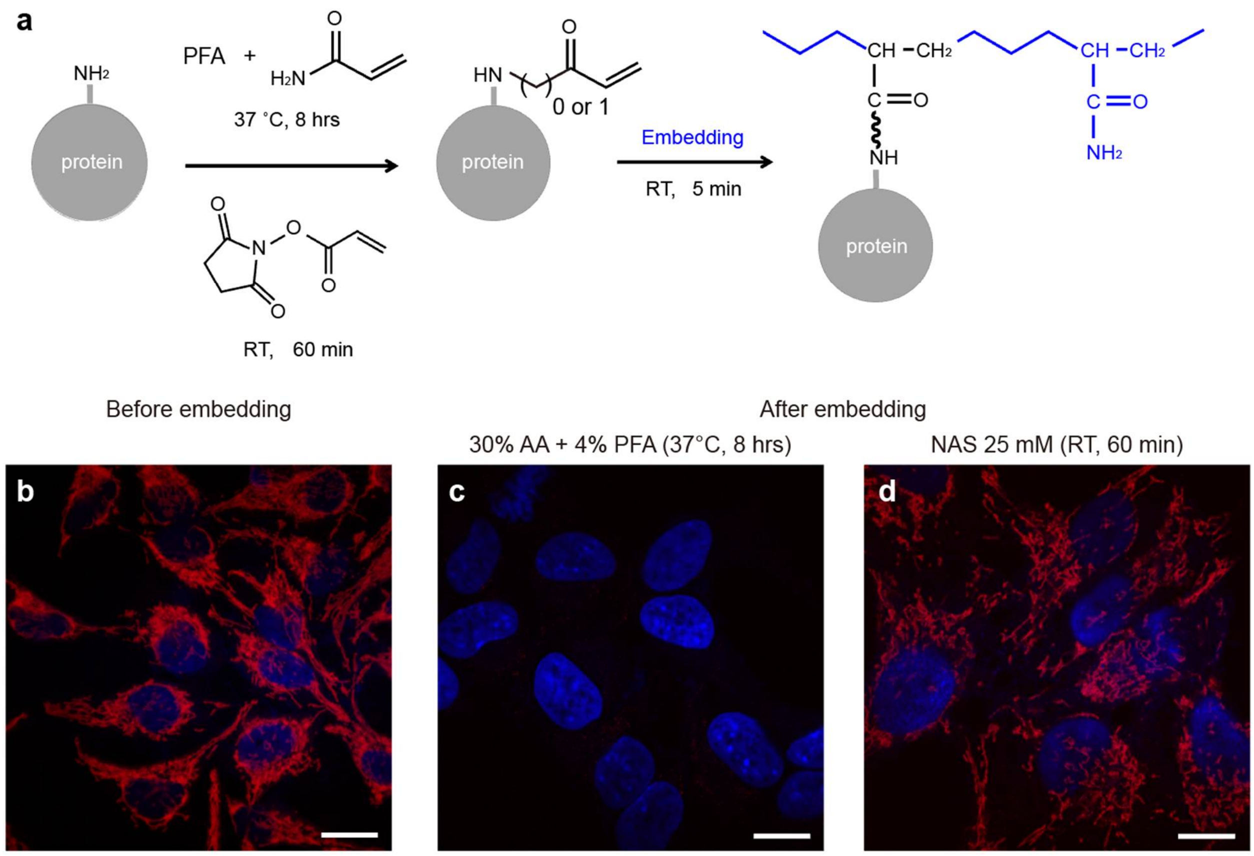

Figure S6. Amine-reactive protein anchors improve retention of proteins.

a) Schematic illustration of two protein anchoring-strategies, one employing the combination of paraformaldehyde (PFA) and acrylamide (AA), and the other employing an amine-reactive protein anchor, *N*-acryloxysuccinimide (NAS). b) HeLa cells were fixed with 3.2% PFA and 0.1% glutaraldehyde (GA) and were stained against TOM20, a protein in the mitochondrial outer membrane (red). Blue indicates nuclear counterstaining with Hoechst 33342. c,d) Fixed HeLa cells were incubated in a 30% AA, 4% PFA solution at 37°C for 8 hrs (c), or in a 25 mM NAS solution for 60 min (d) to create anchoring sites for proteins and the hydrogel network. After gel embedding, however, TOM20 could be successfully stained only in the NAS-treated cells, indicating that NAS can effectively retain endogenous proteins for ZOOM-processing. Scale bars, 20 μm (b-d).

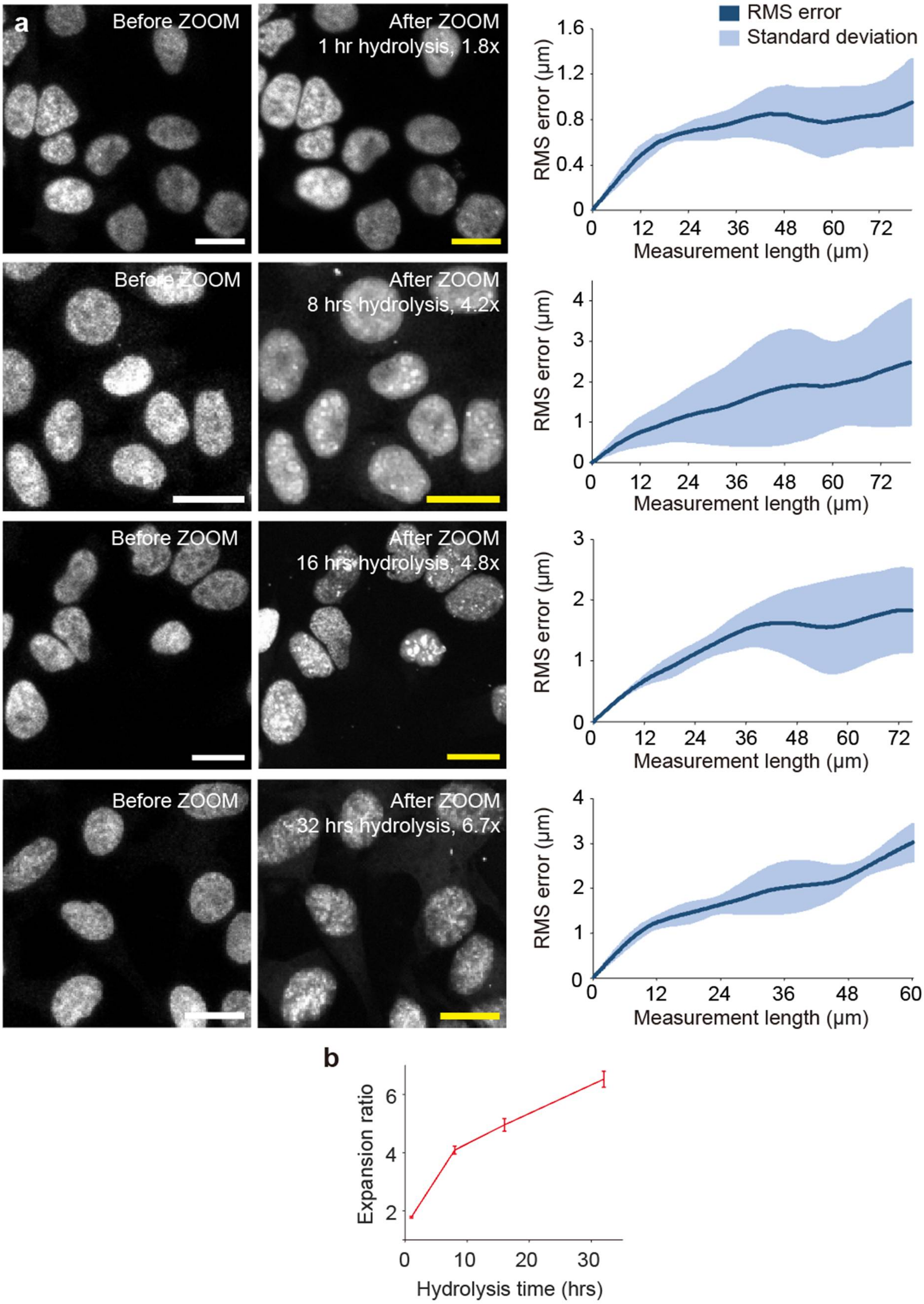

Figure S7. Distortion analysis of ZOOM-processed cultured cells over multiple ZOOM factors.

a) Representative confocal images showing cultured HeLa cells stained with Hoechst 33342 (1 hr, RT, 1:1,000 in PBS) before and after ZOOMing (hydrolysis for 1, 8, 16 or 32 hrs at 80°C) (left), and the averaged RMS error of the images before and after ZOOM processing (*n* = 4 samples for each condition) (right). The average estimated distortion error was less than 5% of the measured length at all conditions, demonstrating isotropic expansion at the multicellular scale, from multiple gels. b) The ZOOM factor increased from 1.8× to 4.1×, 5.0× and 6.5× (for 1, 8, 16 and 32 hrs, respectively; these are averaged values, whereas the ZOOM factors indicated in the images of panel (a) are from the representative samples). Data are mean ± s.d. Scale bars, 20 µm.

Movie S1. ZOOMing into the mouse cortical tissue.

A 500 μm-thick Thy1-eYFP mouse brain section was 8.0-fold expanded with ZOOM. 3D rendering of an expanded cortical tissue volume acquired with confocal microscopy (acquired with 10×, 0.5 NA objective lens; acquisition volume, ~9.0×9.0×1.8 mm^3 post-expansion; estimated lateral resolution 620 nm pre-expansion, 78 nm post-expansion) readily supports tracing of neural processes. Scale bar indicates physical dimensions.

Movie S2. ZOOMing into the cellular microtubule network.

ZOOM-processed HeLa cells were stained for α-tubulin and imaged after ZOOM processing. The video shows fine tubulin structures resolved after 5.2× expansion (acquired with 40×, 1.2 NA objective lens; estimated lateral resolution 168 nm pre-expansion, 32 nm post-expansion).

Table S1. Sample preparation and imaging conditions

Fig	Sample Name	Staining* (antibodies and dyes)<br>*All ZOOM-processed samples were stained following the hydrolysis step.	ZOOM factor	Objective	Voxel size (x, y, z) (adjusted by the ZOOM factor) (μm)	Dimensions (x, y, z) (pixel numbers)	Image volume (x, y, z) (adjusted by expansion factor) (μm)	Pixel dwell time (μs)	Lasers	Master gain	Line averaging	Notes
Fig. 1e-g	Thy1-eYFP H	Alexa 488-conjugated Rabbit anti-GFP	8	W Plan-Apochromat 10×/0.5 M27 75mm	0.415 (0.052)<br>0.415 (0.052)<br>10.000 (1.250)	17503 × 15676 × 181	7266 × 6383 × 1810 (908 × 798 × 226)	0.38	633 nm (10.0%)	750	1	
Fig. 2a	Homer, Basson staining in Thy1-eYFP H (before expansion)	Primary:<br>Guineapig anti-Bassoon<br>Rabbit anti-Homer1<br>Secondary:<br>Donkey anti-Rabbit IgG H&L 594<br>Donkey anti-Guineapig IgG H+L 647	-	C Plan-Apochromat 63× / 1.4 oil DIC UV-VIS-IR M27	0.085<br>0.085<br>0.085	396 × 396 × 26	33.74 × 33.74 × 9.55	1.99	488 nm (2.0%)<br>543 nm (2.0%)<br>633 nm (5.0%)	550<br>550<br>650	16	
	Homer, Basson staining in Thy1-eYFP H (1 hr hydrolysis)	Primary:<br>Chicken anti-GFP<br>Guineapig anti-Bassoon<br>Rabbit anti-Homer1<br>Secondary:<br>Donkey anti-Chicken IgY 549<br>Donkey anti-Rabbit IgG H&L 488<br>Donkey anti-Guineapig IgG H+L 647	2.5	C-Apochromat 40×/1.2 W Corr FCS M27	0.083 (0.033)<br>0.083 (0.033)<br>0.395 (0.158)	1288 × 1288 × 56	106.19 × 106.19 × 21.72 (42.46 × 42.46 × 8.69)	0.3	488 nm (2.0%)<br>543 nm (1.5%)<br>633 nm (2.0%)	650<br>550<br>650	8	Several Z-sections were max intensity-projected to show structure details
	Homer, Basson staining in Thy1-eYFP H (3 hrs hydrolysis)	Primary:<br>Chicken anti-GFP<br>Guineapig anti-Bassoon<br>Rabbit anti-Homer1<br>Secondary:<br>Donkey anti-Chicken IgY 549<br>Donkey anti-Rabbit IgG H&L 488<br>Donkey anti-Guineapig IgG H+L 647	3.7	C-Apochromat 40×/1.2 W Corr FCS M27	0.083 (0.022)<br>0.083 (0.022)<br>0.404 (0.109)	1288 × 1288 × 120	106.19 × 106.19 × 48.06 (28.7 × 28.7 × 12.99)	0.3	488 nm (2.0%)<br>543 nm (1.5%)<br>633 nm (4.0%)	650<br>550<br>600	8	Several Z-sections were max intensity-projected to show structure details
	Homer, Basson staining in Thy1-eYFP H (12 hrs hydrolysis)	Primary:<br>Chicken anti-GFP<br>Guineapig anti-Bassoon<br>Rabbit anti-Homer1<br>Secondary:<br>Donkey anti-Chicken IgY 549<br>Donkey anti-Rabbit IgG H&L 488<br>Donkey anti-Guineapig IgG H+L 647	5.5	C-Apochromat 40×/1.2 W Corr FCS M27	0.100 (0.018)<br>0.100 (0.018)<br>0.395 (0.072)	1068 × 1068 × 61	106.17 × 106.17 × 23.69 (19.30 × 19.30 × 4.31)	0.37	488 nm (3.0%)<br>543 nm (7.0%)<br>633 nm (5.0%)	750<br>800<br>650	8	Several Z-sections were max intensity-projected to show structure details
Fig. 2f	Lectin staining of mouse cortical tissue (before expansion)	DyLight 649-conjugated tomato lectin	-	W Plan-Apochromat 10×/0.5 M27 75mm	0.415<br>0.415<br>3.681	3789 × 1946 × 28	1572.86 × 807.68 × 103.07	0.38	633 nm (2.0%)	550	2	For the purpose of confirming minimal distortion over the multi-round ZOOM procedure, we collected the images with quality and volume sufficiently high and large enough for the distortion analysis. Accordingly, acquisition
	Lectin staining of mouse cortical tissue (15min hydrolysis)	DyLight 649-conjugated tomato lectin	2.4	W Plan-Apochromat 10×/0.5 M27 75mm	0.415 (0.173)<br>0.415 (0.173)<br>8.065 (3.36)	9011 × 5530 × 35	3740.85 × 2295.52 × 282.28 (1558.7 × 956.5 × 117.6)	0.38	633 nm (3.0%)	650	2	
	Lectin staining of mouse cortical tissue	DyLight 649-conjugated tomato lectin	4.5	W Plan-Apochromat 10×/0.5 M27 75mm	0.830 (0.184)<br>0.830 (0.184)<br>10.000	7475 × 4710 × 52	6206.40 × 3910.88 × 520 (1379.2 × 869.1 ×	0.77	633 nm (2.0%)	550	2	

	(2 hrs hydrolysis)				(2.22)		115.6)					pixel sizes were larger than the Nyquist pixel size. The imaging volume is variable due to the empty spaces imaged outside the sample.
	Lectin staining of mouse cortical tissue<br>(9 hrs hydrolysis)	DyLight 488-conjugated tomato lectin	5.7	W Plan-Apochromat 10×/0.5 M27 75mm	0.830 (0.146)<br>0.830 ((0.146)<br>15.000 (2.63)	9318 × 5632 × 50	7736.75 × 4676.06 × 750 (1357.3 × 820.3 × 131.6)	0.77	488 nm (5.0%)	850	2	
	Lectin staining of mouse cortical tissue<br>(18 hrs hydrolysis)	DyLight 649-conjugated tomato lectin	6.7	W Plan-Apochromat 10×/0.5 M27 75mm	1.384 (0.207)<br>1.384 (0.207)<br>15.000 (2.24)	6042 × 3738 × 50	8360.22 × 5172.00 × 750 (1247.8 × 771.94 × 111.94)	1.02	633 nm (5.0%)	700	2	
Fig. 3b	Microtubule	Primary:<br>Mouse anti-alpha tubulin<br>Secondary:<br>Goat anti-mouse IgG H&L Alexa 647	4.6	C Plan-Apochromat 63×/1.4 oil DIC UV-VIS-IR M27	0.11 (0.024)<br>0.11 (0.024)<br>0.49 (0.107)	1220 × 1220 × 61	134.95 × 134.95 × 29.69 (29.34 × 29.34 × 6.45)	0.64	633 nm (10.0 %)	900	4	Several Z-sections were max intensity-projected to show structure details
Fig. 3f	Mitochondria	Alexa 488-conjugated Rabbit anti-TOMM20	3.1	C Plan-Apochromat 63×/1.4 oil DIC UV-VIS-IR M27	0.09 (0.029)<br>0.09 (0.029)	1584 × 1584	134.95 × 134.95 (43.53 × 43.53)	2.65	488 nm (30.0 %)	800	16	Imaging conditions were the same for both pre- and post-ZOOM images
Fig. 3g	Centriole (before expansion)	Primary:<br>Rabbit anti-CEP164<br>Secondary:<br>Donkey anti-rabbit IgG H&L Alexa 647	-	C Plan-Apochromat 63×/1.4 oil DIC UV-VIS-IR M27	0.09<br>0.09<br>0.40	1584 × 1584 × 34	134.95 × 134.95 × 13.12	0.5	488 nm (10.0 %)<br>633 nm (5.0 %)	800<br>900	8	Several Z-sections were max intensity-projected to show structure details
	Centriole (after expansion)	Primary:<br>Rabbit anti-CEP164<br>Mouse anti-Centrin<br>Secondary:<br>Donkey anti-rabbit IgG H&L Alexa 647<br>Goat anti-mouse IgG H&L Alexa 488	6.3	C-Apochromat 40×/1.2 W Corr FCS M27	0.10 (0.016)<br>0.10 (0.016)<br>0.48 (0.076)	712 × 712 × 13	70.85 × 70.85 × 5.74 (11.25 × 11.25 × 0.91)	1.1	488 nm (8.0 %)<br>633 nm (8.0 %)	900<br>900	16	Several Z-sections were max intensity-projected to show structure details
Fig. 3h	Centriole (after ZOOM + Airy scan)	Primary:<br>Rabbit anti-CEP164<br>Secondary:<br>Goat anti-rabbit IgG H&L Alexa 647	6.3	C-Apochromat 40×/1.2 W Corr FCS M27	0.06 (0.010)<br>0.06 (0.010)<br>0.27 (0.043)	1076 × 1076 × 19	69.30 × 69.30 × 4.86 (11.00 × 11.00 × 0.77)	0.96	633 nm (10.0 %)	900	8	Airyscan-processed z-sections were max intensity-projected to show structure details
Fig. 3i	Centriole (SIM)	Primary:<br>Rabbit anti-CEP164<br>Secondary:<br>Goat anti-rabbit IgG H&L Alexa 647	-	Olympus PlanApo N 60x/1.42 Oil	0.04<br>0.04	512 x 512	20.48 x 20.48	-	633 nm (10.0 %)	Exposure time 20ms	-	
Fig. 4b-e	Homer and Bassoon staining in PV::tdTomato and virus injected CaMKII-eYFP	Primary:<br>Rabbit anti-Homer1<br>Goat anti-tdTomato<br>Chicken anti-GFP<br>Guineapig anti-Bassoon<br>Secondary:<br>Donkey anti-Goat H&L 405<br>Donkey anti-Chicken IgY 549<br>Donkey anti-Rabbit H&L 488<br>Donkey anti-Guineapig H+L 647	4	C-Apochromat 40×/1.2 W Corr FCS M27	0.082 (0.021)<br>0.082 (0.021)<br>0.622 (0.156)	2580 × 2580 × 96	212.55 × 212.55 × 59.05 (53.14 × 53.14 × 14.76)	0.3	405 nm (4.0%)<br>488 nm (2.0%)<br>543 nm (3.0%)<br>633 nm (3.0%)	750<br>600<br>850<br>750	8	Several Z-sections were max intensity-projected to show structure details
Fig. 4i-l	Thy1-eYFP H<br>Light-sheet	Alexa 488-conjugated Rabbit anti-GFP	6	EC Plan-Neofluar 5×/0.16	0.323 (0.054)<br>0.323 (0.054)<br>2.4 (0.4)	15168 × 17907 × 770	4894 × 5778 × 1847 (816 × 963 × 308)	-	488 nm (10.0%)	-	-	Acquired with Zess Z1 Light-sheet microscopy
Fig.	*C. elegans*	Alexa 488-conjugated	3.1	W Plan-	0.119	7850 ×	935.21 ×	0.2	488 nm	670	8	Several Z-

5b-f	*mec-7p*::GFP	Rabbit anti-GFP		Apochromat 20×/1.0 Corr DIC M27 75mm	(0.038)<br>0.119<br>(0.038)<br>0.778<br>(0.251)	6333 × 274	754.55 × 213.24 (301.68 × 243.30 × 68.79)	4	(3.0%)			sections were max intensity-projected to show structure details
Fig. 5h	*E. coli* K12 (before ZOOM)	Primary:<br>Mouse anti-ATPB antibody<br>Secondary:<br>Goat anti-mouse IgG H&L Alexa 488	-	C Plan-Apochromat 63x/1.4 oil DIC UV-VIS-IR M27	0.09<br>0.09	316 × 316	26.99 × 26.99	0.79	543 nm (2.0 %)	650	16	
	E. coli K12 (after ZOOM)	Primary:<br>Mouse anti-ATPB antibody<br>Secondary:<br>Goat anti-mouse IgG H&L Alexa 488	3.5	C Plan-Apochromat 63×/1.4 oil DIC UV-VIS-IR M27	0.09<br>(0.026)<br>0.09<br>(0.026)	1024 × 1024	29.99 × 29.99 (8.57 × 8.57)	1.02	488 nm (4.5 %)	750	8	
Fig. 5j	PD human	SYTO-16<br>Primary:<br>Mouse anti-phospho-alpha-Synuclein Phospho Ser129<br>Secondary :<br>Donkey anti-mouse IgG H&L 647	3.7	W Plan-Apochromat 10×/0.5 M27 75mm	0.265<br>(0.066)<br>0.265<br>(0.066)<br>2.4 (0.6)	3212 × 3212 × 43	850.19 × 850.19 × 156.53 (212.55 × 212.55 × 38.38)	0.24	488 nm (2.0%)<br>633 nm (5.0%)	500<br>800	4	
Fig. 5k	PD human	SYTO-16<br>Primary:<br>Mouse anti-phospho-alpha-Synuclein Phospho Ser129<br>Secondary :<br>Donkey anti-mouse IgG H&L 647	3.7	C-Apochromat 40×/1.2 W Corr FCS M27	0.109<br>(0.027)<br>0.109<br>(0.027)<br>0.478<br>(0.120)	1944 × 1944 × 176	212.44 × 212.44 × 83.72 (53.11 × 53.11 × 20.93)	0.4	488 nm (2.0%)<br>633 nm (5.0%)	500<br>800	4	
Fig. S3	Thy1-eYFP H Dendrite 3.5×	Alexa 488-conjugated Rabbit anti-GFP	3.5	C-Apochromat 40×/1.2 W Corr FCS M27	0.100<br>(0.029)<br>0.100<br>(0.029)<br>0.520<br>(0.149)	1068 × 1068 × 56	106.27 × 106.27 × 28.375	0.37	488 nm (3.0 %)	750	16	-
	Thy1-eYFP H Dendrite 6.1×	Alexa 488-conjugated Rabbit anti-GFP	6.1	C-Apochromat 40×/1.2 W Corr FCS M27	0.100<br>(0.016)<br>0.100<br>(0.016)<br>0.520<br>(0.085)	712 × 712 × 112	70.85 × 70.85 × 57.63	0.48	488 nm (8.0 %)	750	16	-
Fig. S6	Cultured HeLa Cell	Hoechst 33342	6.6	W Plan-Apochromat 10×/0.5 M27 75mm	0.22<br>(0.033)<br>0.22<br>(0.033)	3872 × 3872	850.19 × 850.19 (128.82 × 128.82)	0.27	405 nm (10.0 %)	800	16	Imaging conditions were the same for both pre- and post-ZOOM images
Fig. S7a	Cultured HeLa Cell nucleus staining before expansion	DAPI	-	W Plan-Apochromat 10x/0.5 M27 75mm	0.42<br>0.42<br>6.69	Various by samples	Various by samples	0.38	405 nm (2.0 %)	550	2	
	Cultured HeLa Cell nucleus staining (1 hr hydrolysis)	DAPI	1.8	W Plan-Apochromat 10x/0.5 M27 75mm	0.42 (0.23)<br>0.42 (0.23)<br>6.69 (3.72)	Various by samples	Various by samples	0.38	405 nm (2.0 %)	750	4	
	Cultured HeLa Cell nucleus staining (8 hrs hydrolysis)	DAPI	4.2	W Plan-Apochromat 10x/0.5 M27 75mm	0.42 (0.10)<br>0.42 (0.10)<br>8.83 (2.10)	Various by samples	Various by samples	0.38	405 nm (4.0 %)	750	4	
	Cultured HeLa Cell nucleus staining (16 hrs hydrolysis)	DAPI	4.8	W Plan-Apochromat 10x/0.5 M27 75mm	0.42 (0.09)<br>0.42 (0.09)<br>7.20 (1.50)	Various by samples	Various by samples	0.38	405 nm (5.0 %)	850	4	
	Cultured	DAPI	6.7	W Plan-	0.83 (0.12)	Various	Various	1.0	405 nm	950	4	

	HeLa Cell nucleus staining (32 hrs hydrolysis)			Apochromat 10x/0.5 M27 75mm	0.83 (0.12) 11.92 (1.78)	by samples	by samples	2	(8.0 %)			
